# Supplementary material for: Molecular pixelation: spatial proteomics of single cells by sequencing
Source: Nat Methods. 2024 May 8;21(6):1044–52. doi: 10.1038/s41592-024-02268-9 (PMC11166577; doi:10.1038/s41592-024-02268-9)
Supplement: Supplementary file 1 — Supplementary Figures 1–9, Supplementary Protocol. [file 41592_2024_2268_MOESM1_ESM.pdf]

nature methods

Article

https://doi.org/10.1038/s41592-024-02268-9

Molecular pixelation: spatial proteomics of single cells by sequencing

In the format provided by the authors and unedited

Supplementary Figure S1.

Scanning Electron Microscopy (SEM) of DNA-pixels. SEM imaging performed by Swedish National Microscopy Infrastructure, NMI (VR-RFI 2016-00968). RCA-products collapse into spheres which has previously been shown by SEM (Deng *et al* DNA-Sequence-Encoded Rolling Circle Amplicon for Single-Cell RNA Imaging. Chem 4, 1373–1386, June 14, 2018). No DNA-pixels were found over the size of 100 nm, showing similar size as previously reported for RCA products (Wu D, *et al* Profiling surface proteins on individual exosomes using a proximity barcoding assay. *Nat Commun.* Aug 26;10(1):3854. 2019).

DNA Pixel behavior and area coverage when bound to a cell by hybridization likely differs from the SEM measurement, resulting in only estimates of spatial resolution possible. One could assume that if the ~1,500 DNA pixels which bind to a cell, also covers the entire cell surface area of a typical lymphocyte of 92 um^2 (Majstoravich S. *et al* Lymphocyte microvilli are dynamic, actin-dependent structures that do not require Wiskott-Aldrich syndrome protein (WASp) for their morphology Blood. Sep 1;104(5):1396-403. 2004), the average area covered by one DNA-pixel would be 0.06 um^2, representing a diameter of up to 280 nm and being an upper limit on resolution, while the lower limit can be estimated at the SEM based DNA Pixel size below 100 nm. Further investigations are ongoing to determine a more precise spatial resolution of MPX.

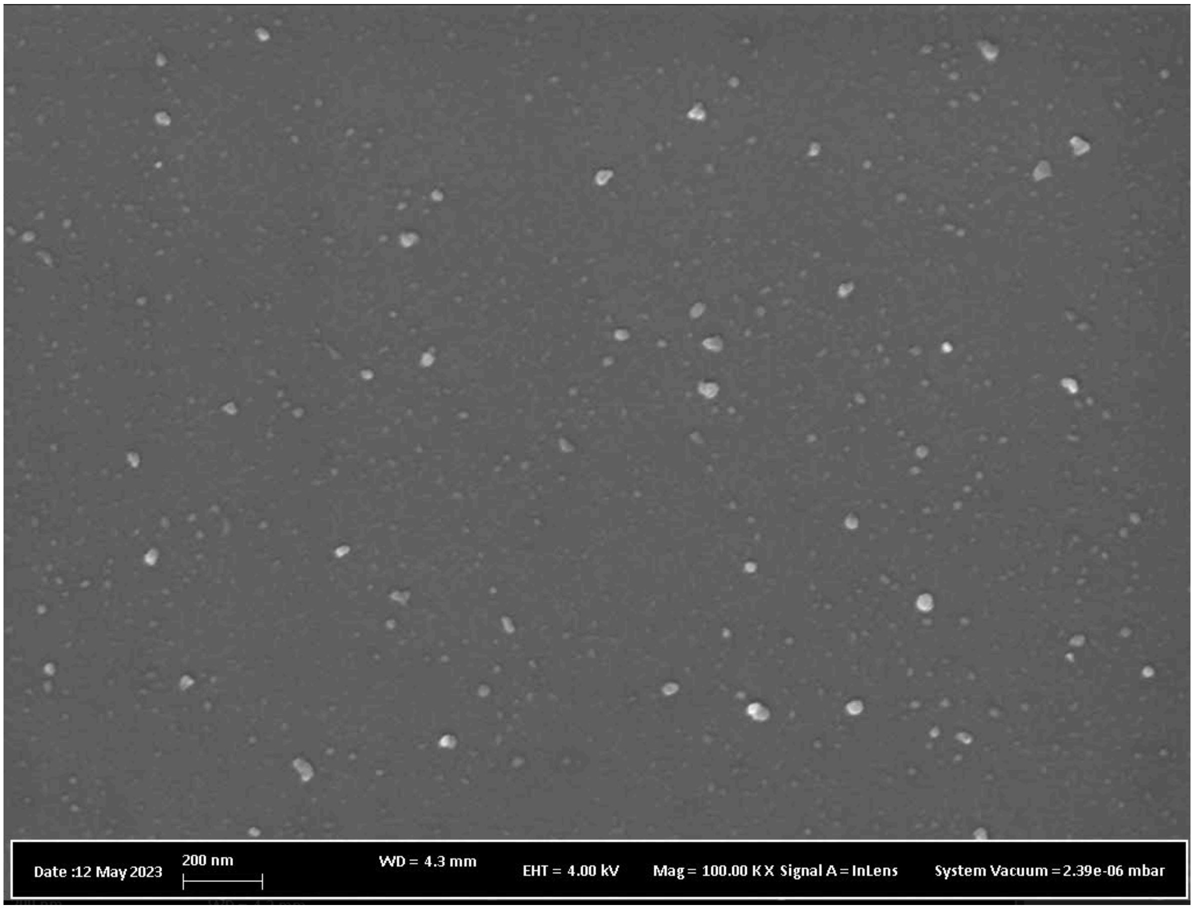

Supplementary Figure S2.

Cell input range linearity. One sample was processed with Molecular pixelation and cells were counted prior to PCR reaction. 200, 500 or 1000 cells were input to separate PCR reactions, and carried forward to sequencing. The number of detected cells after processing the data through the data analysis pipeline correlated well with the number of input cells into PCR (R^2 value of 0.99). The number of detected cells were 161, 517 and 1054 for the inputs of 200, 500 and 1000 cells respectively. Variability from counting, dilution and aliquoting cells into PCR likely contributed to some of the deviation from expected input.

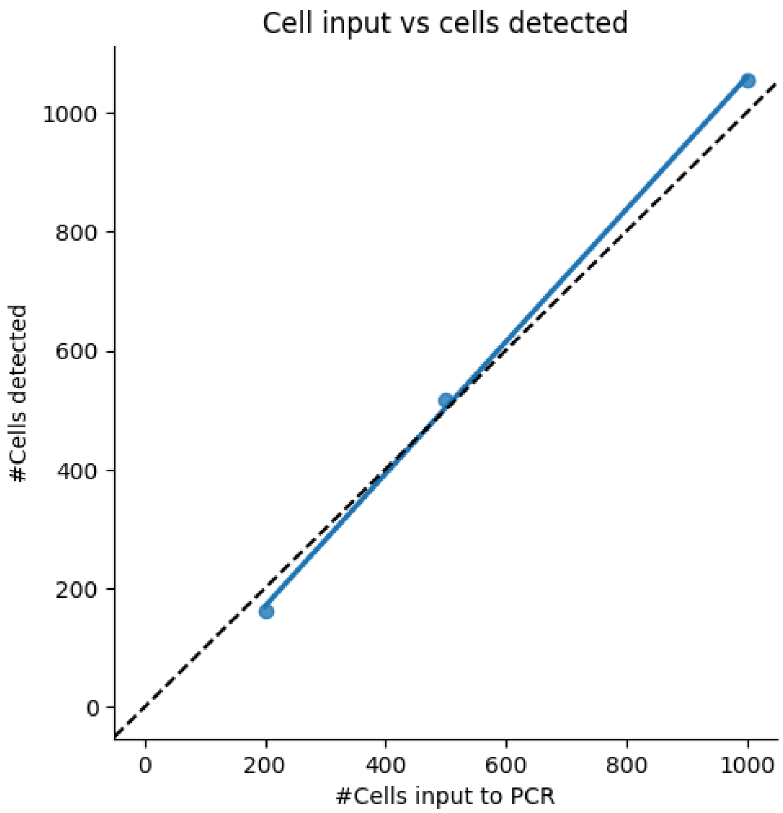

Supplementary Figure S3.

Comparison of cell type fractions and dynamic ranges for MPX and flow cytometry. Fixed PBMC cells were split into two fractions. One fraction was processed with MPX across 4 separate reactions and data combined for a total of 2025 cells identified in data analysis. Cell fractions were estimated by gating sub-populations from the clr-transformed count data. $CD3^+$, $CD19^+$, $CD16^+$, $CD14^+$ fractions were estimated from the $CD45^+$ population. $CD4^+$ and $CD8^+$ fractions were estimated from the ($CD45^+$, $CD3^+$) population. Flow cytometry was performed on the second PBMC fraction in 4 separate reactions using fluorescently-labeled antibodies of the same clones as used in MPX, in the following combinations: (CD45, CD3, CD4, CD8), (CD45, CD19), (CD45, CD14) or (CD45, CD16). Dynamic ranges for MPX were calculated from the median raw count per cell per gated sub-population for MPX data, and from the median fluorescent intensity values for the flow cytometry data. The estimated fractions and dynamic ranges for each subpopulation is listed in Table S2. Scatterplots and gating cutoffs for MPX clr-counts per cell (left) and for flow cytometry intensity values (right) for A) $CD3^+$, $CD4^+$, $CD8^+$ cell fractions, and B) $CD19^+$, $CD14^+$ and $CD16^+$ fractions.

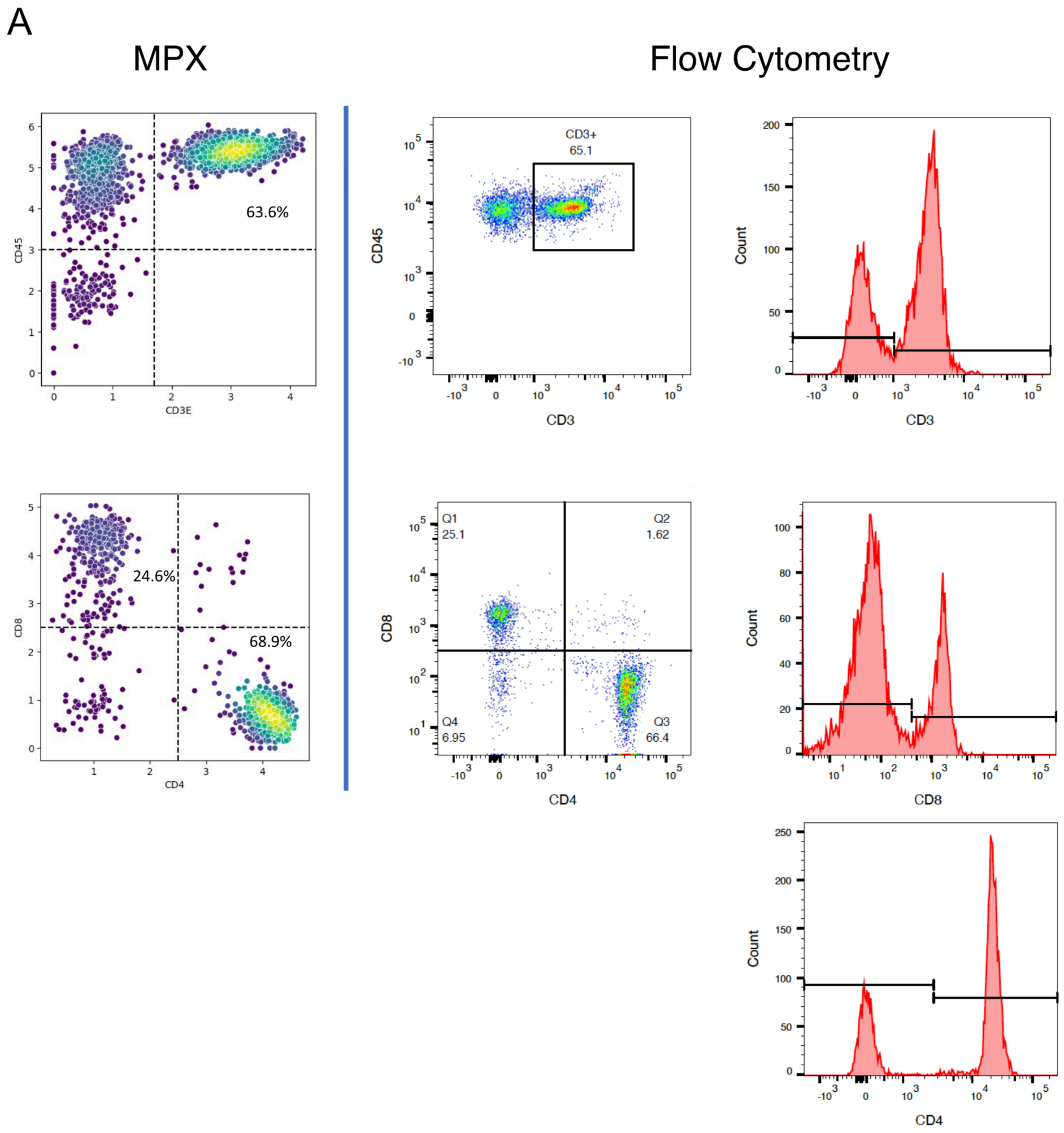

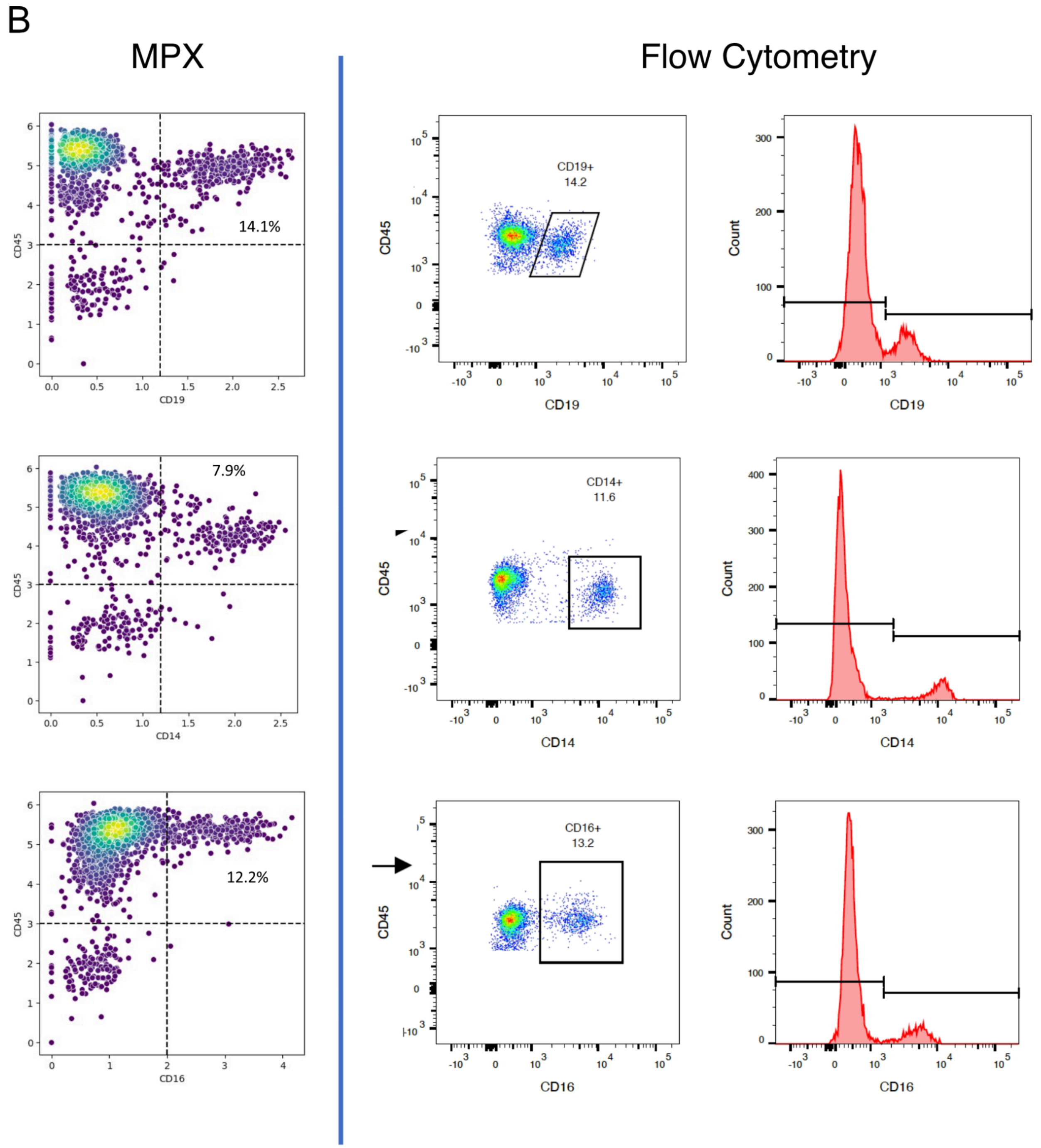

B
MPX
Flow Cytometry
14.1%
7.9%
12.2%
CD45
CD19
CD14
CD16
CD19+
14.2
CD14+
11.6
CD16+
13.2
Count

Supplementary Figure S4.

Heatmap of scaled counts of all 80 markers (x-axis; including the 4 control markers) across all cells after filtering (y-axis; n = 814). The two tracks to the left of the heatmap denote the source sample and the cell type annotation of the components. The color corresponds to CLR transformed counts.

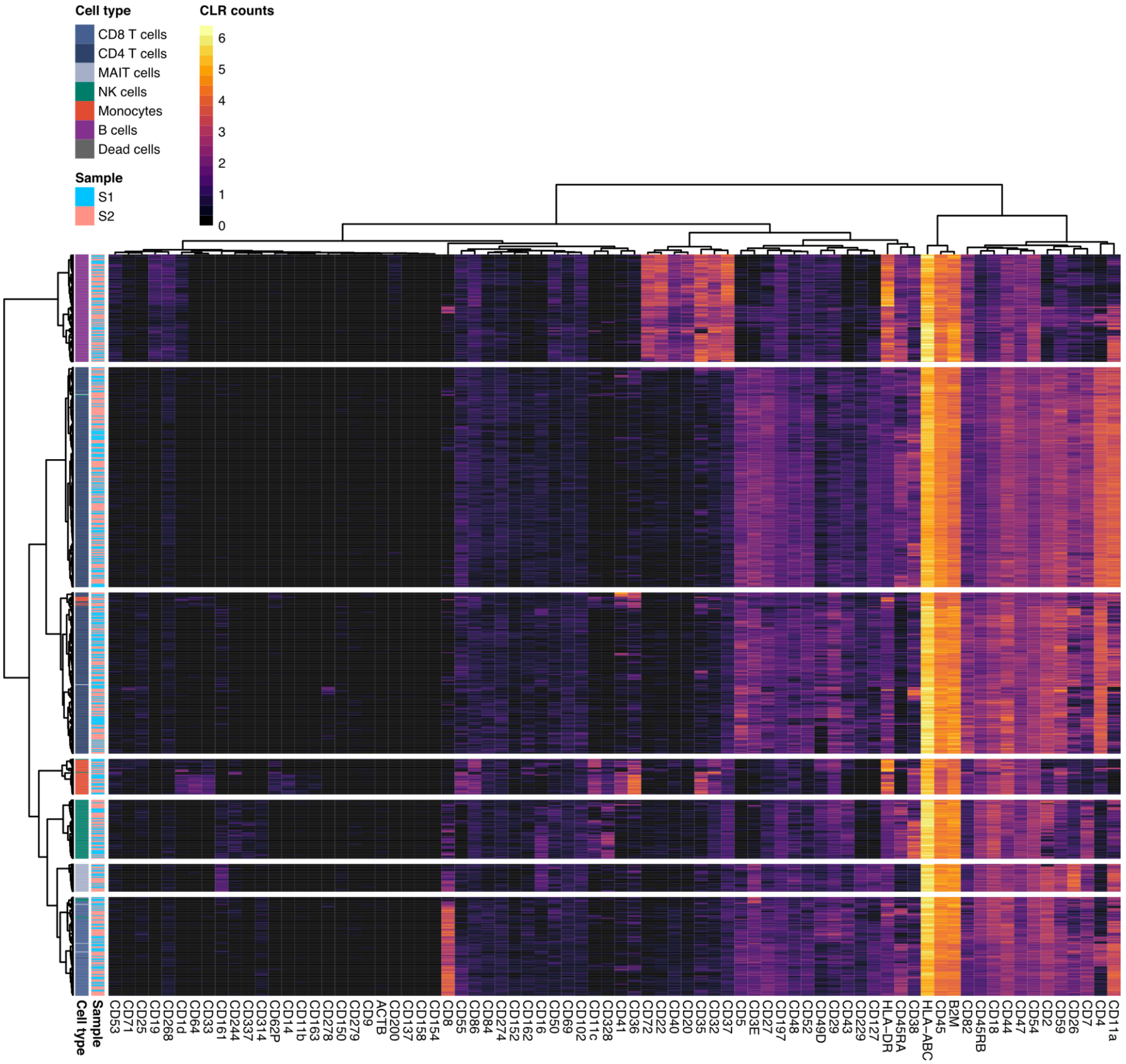

Supplementary Figure S5.

Violin plot showing the distribution of relative counts (%) for isotype markers in unstimulated cells. The median percentage is marked.

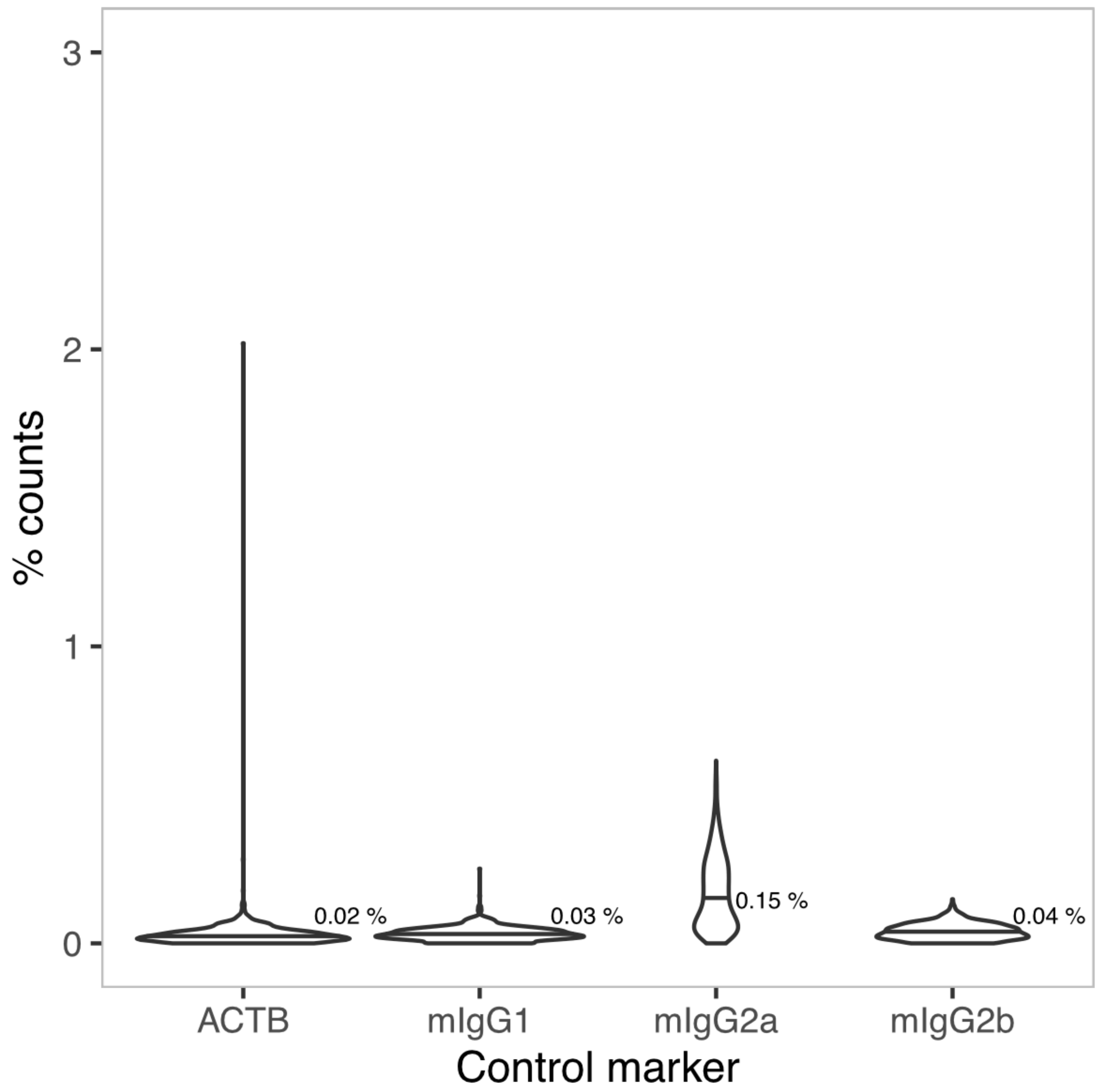

Supplementary Figure S6.

Volcano plots for the differential polarization analysis (two-sided Wilcoxon Rank Sum test) for A) T cells in PBMC stimulated by CD3 capping and B) Raji cells stimulated with Rituximab.

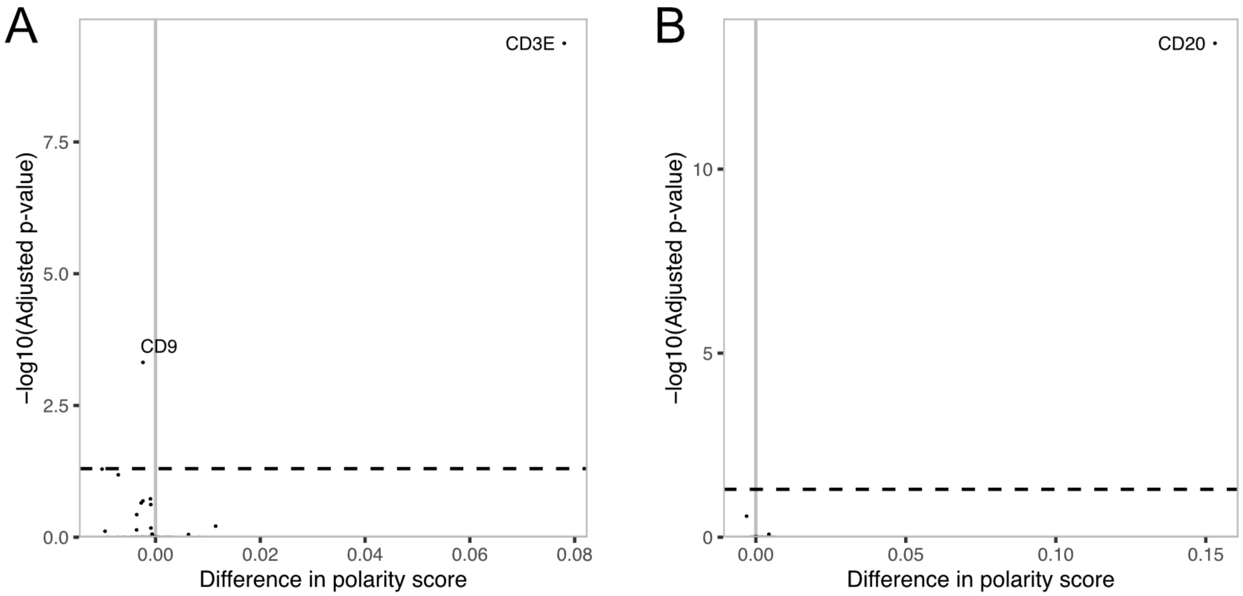

Supplementary Figure S7.

Examples of 3 approaches for 3D graph visualization layouts for proteins CD50, CD162 and CD3 from one selected chemokine-stimulated cell. Left: Layout obtained from force directed layout algorithm Kamada Kawai (kk). Middle: Projection of coordinates from kk layout onto unit sphere. Right: Heat map interpolation of count density onto a sphere surface. See methods for further details. The plots show a clustered or polarized protein arrangement of CD50 and CD162 in the same location, thus also showing colocalization of these markers, while CD3 is randomly distributed across the cell graph.

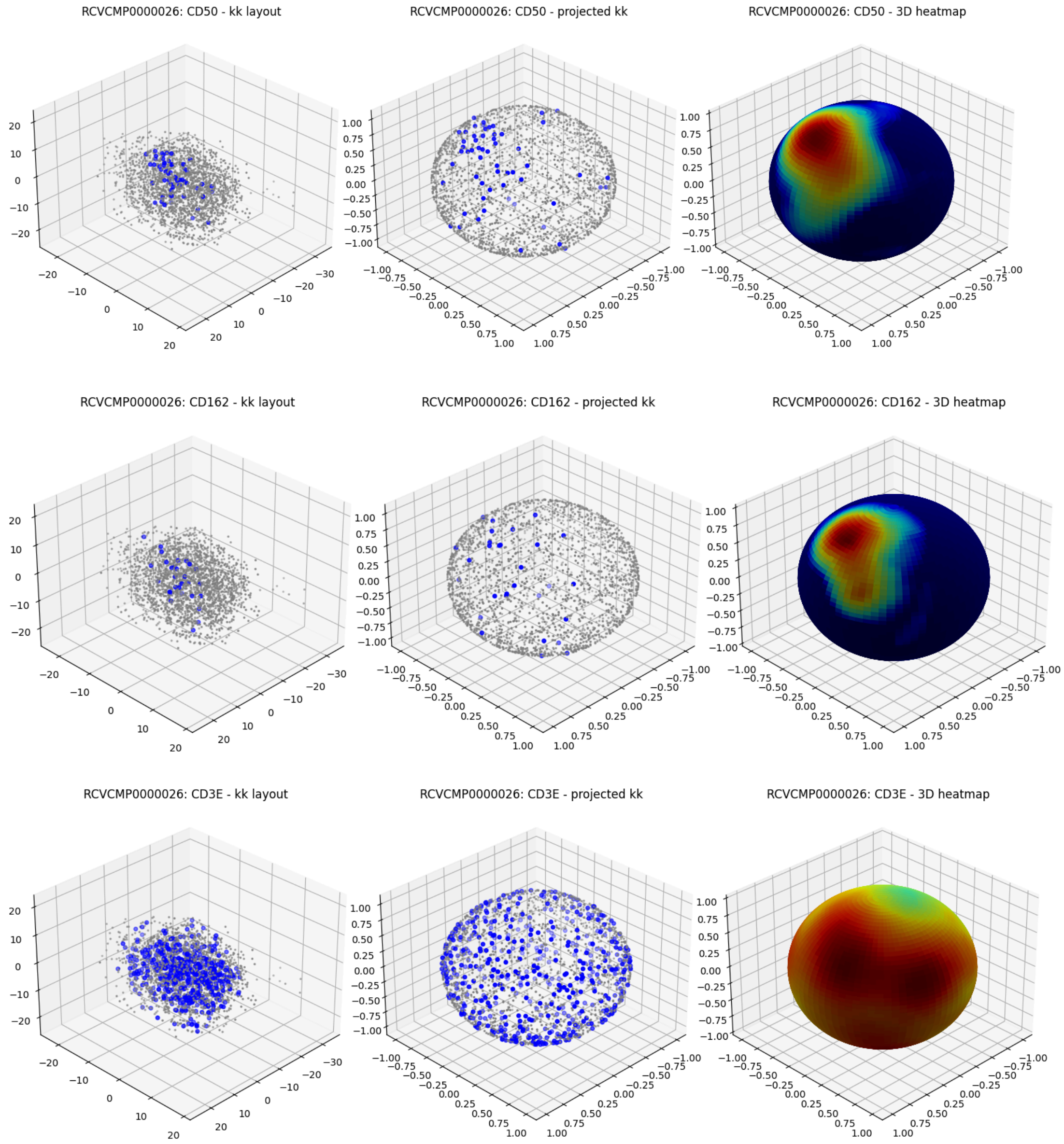

Supplementary Figure S8.

Edge rank plot for the two replicates of healthy PBMC, analogous to the “Barcode Rank Plot” often used to visualize cell calling in scRNAseq. The y-axis displays the number of edges (UMIs) per cell, which are ranked from highest to lowest along the x-axis. The two dashed lines mark the hard cutoffs for filtering the smallest and largest cells.

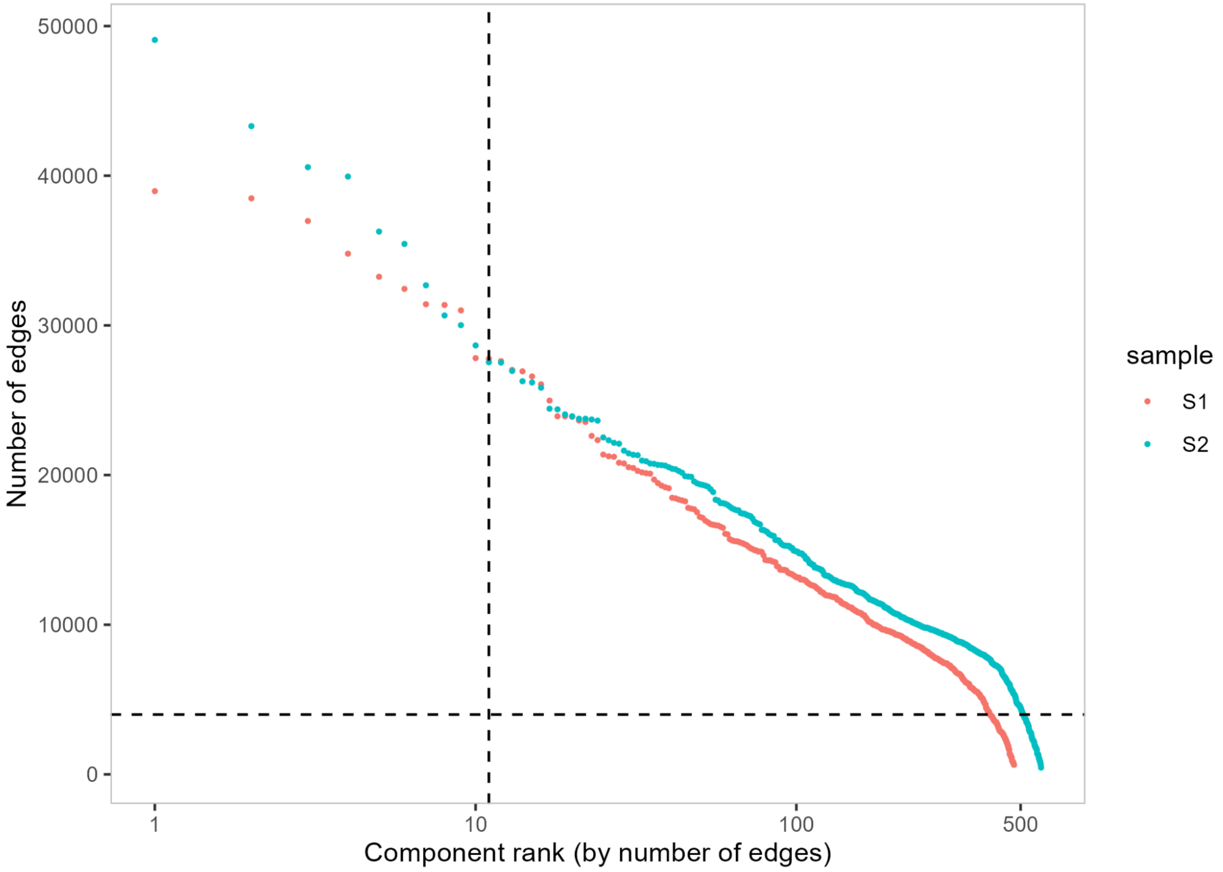

Supplementary Figure S9.

Distribution of abundance of some canonical markers and cutoffs used to gate T cells from PBMC. T cells were gated by the following cutoffs: CD3E > 0.9, CD4 > 1.8 or CD8 > 1.5, CD19 < 0.5, CD20 < 1.1, CD14 < 0.5.

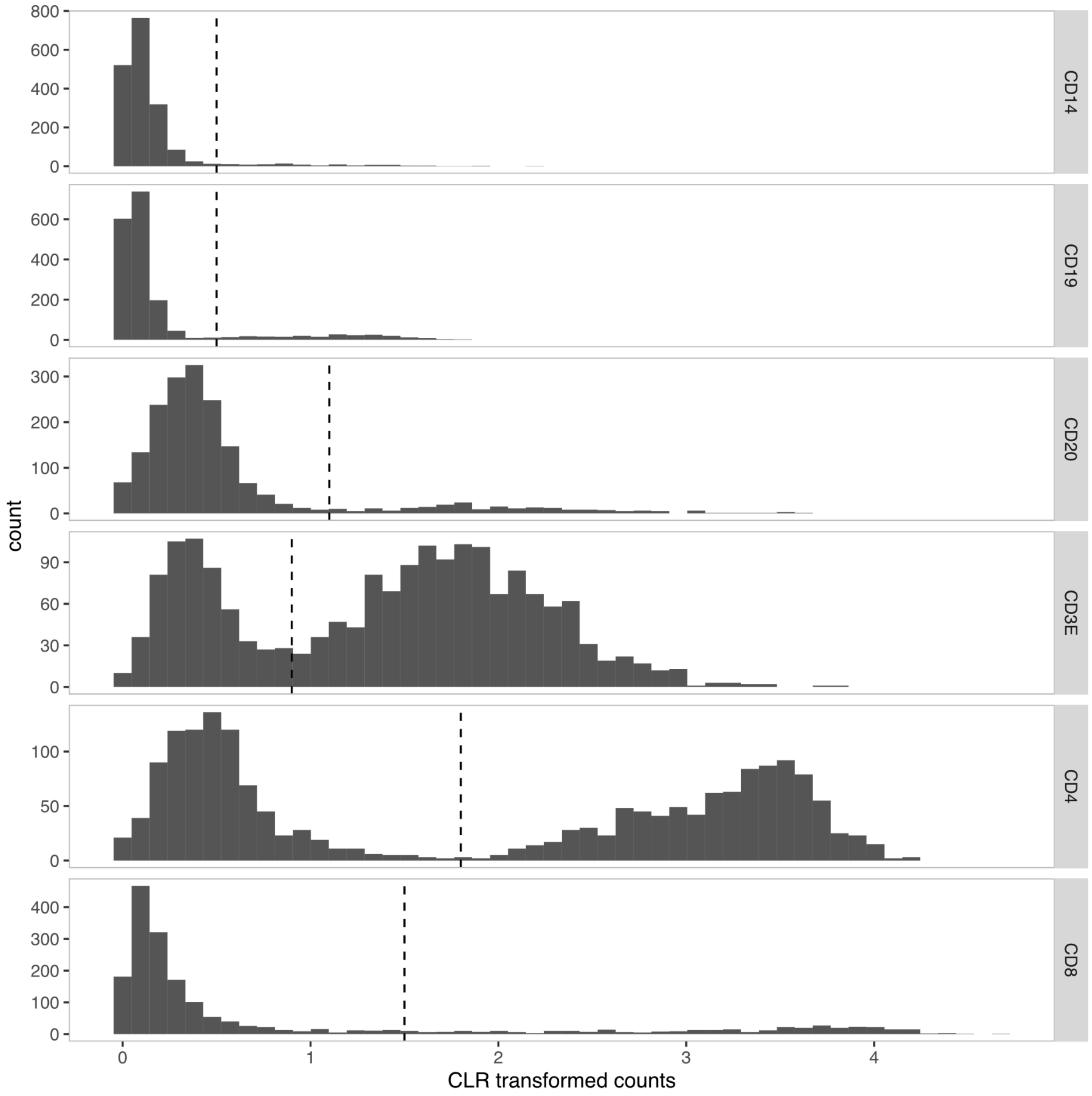

Supplementary MPX PROTOCOL

Table of Contents

STEP 1 - Cell preparation 1

Cell preparation 1

Cell fixation and blocking 1

OPTIONAL: Freezing and thawing of fixed cells 3

Freezing of PFA-fixed cells 3

Thawing of frozen cells 3

STEP 2 - Antibody-Oligo Conjugate (AOC) binding 4

AOC binding 4

STEP 3.1 - Molecular Pixelation (Pixelation A and B) 6

DNA-Pixel A hybridization 6

Gap-fill ligation step 1 7

Pixel A remover 8

DNA-pixel B hybridization 9

Gap-fill ligation step 2 10

STEP 3.2 - Molecular Pixelation (Exonuclease and PCR) 11

Exonuclease treatment 11

Sample index PCR 12

STEP 4 - NGS preparation 14

AMPure XP beads clean-up 14

Appendix 1, MPX Buffer mix preparation 16

STEP 1 - Cell preparation

During Step 1 of the workflow, live cell suspensions are fixated using paraformaldehyde (PFA) and blocked using the Blocking solution.

Cell preparation

Note Check visually for cell aggregates or debris as these can contribute to inaccurate cell counting. If needed, filter the cell suspension using a cell strainer to remove large aggregates.

Note It is important to pipette the cell suspension gently throughout this part of the protocol.

A. Count the cells using either automated cell counter (e.g., Countess II Automated Cell Counter), hemocytometer or other cell counting device, aiming at >95% viability.

B. For each reaction, transfer 500 000 - 1 million cells in <150 µl 1x PBS to separate PCR tubes.

C. Centrifuge at 400 rcf for 4 min at 4°C.

D. Carefully discard supernatant without disturbing the cell pellet, leaving behind 25 µl supernatant to preserve the pellet.

E. Add 125 µl of 1x PBS on top of the 25 µl cell suspension, gently pipette up and down 10 times.

F. Centrifuge at 400 rcf for 4 min at 4°C.

G. Carefully discard 125 µl supernatant without disturbing the cell pellet, leaving behind 25 µl supernatant to preserve the pellet.

H. Add 65 µl of 1x PBS on top of the 25 µl cell suspension, gently pipette up and down 10 times or until the cells are uniformly suspended and proceed to Cell fixation and blocking.

Cell fixation and blocking

Note Use the necessary precautions when handling PFA solution since it is a CMR substance (Carcinogenic, Mutagenic, or toxic to Reproduction).

Note Prepare a fresh solution of 2% v/v PFA solution in 1xPBS. A volume of 100 µl is needed per reaction (90µl + extra). Use the solution within 2 hours, and store in dark until use.

A. Add 90 µl of the 2% PFA solution to each reaction and pipette up and down 10 times or until the cells are uniformly suspended.

B. Centrifuge at 700 rcf for 4 min at RT.

C. From each 180 µl fixation reaction, carefully discard 155 µl supernatant without disturbing the cell pellet, leaving behind 25 µl of supernatant.

D. Add 125 µl of **Wash Buffer** on top of the 25 µl cell suspension and pipette up and down 10 times.

E. Centrifuge at 700 rcf for 4 min at RT.

F. Carefully discard 135 µl supernatant without disturbing the cell pellet, leaving behind 15 µl of supernatant.

G. Add 165 µl **Blocking Buffer** and resuspend the pellet by pipetting up and down 10 times.

H. Incubate for 15 min at 4°C.

I. Centrifuge at 700 rcf for 4 min at RT.

J. Carefully discard 155 µl supernatant without disturbing the cell pellet, leaving behind 25 µl supernatant to preserve the pellet.

K. Add 125 µl of **Wash Buffer** on top of the 25 µl cell suspension and pipette

L. Centrifuge at 700 rcf for 4 min at RT.

M. Carefully discard 125 µl supernatant without disturbing the cell pellet, leaving behind 25 µl supernatant to preserve the pellet.

N. Add 125 µl of 1x PBS on top of the 25 µl cell suspension.

O. Optional: Determine the cell concentration after PFA fixation, using either hemocytometer or other cell counting device (e.g. Countess II Automated Cell Counter).

Note At this step, cells can be stored at 4°C in 1xPBS for up to 5 days or frozen at -80°C (see optional below) until proceeding with AOC binding.

OPTIONAL: Freezing and thawing of fixed cells

This is an optional step, if planning to do AOC binding within 5 days, please proceed to *STEP 2 - Antibody-Oligo Conjugate (AOC) binding*.

The fixed and blocked cells can be frozen in -80°C for longer storage, up to 3 months. This part of the protocol describes the freezing and thawing procedure.

Freezing of PFA-fixed cells

Note Prepare a fresh freezing solution of 5% DMSO and 95% FBS. A volume of 600 µl per reaction is needed (500 µl + extra).

A. Centrifuge at 700 rcf for 4 min at RT and remove 125 µl supernatant without disturbing the cell pellet, leaving behind 25 µl.

B. Add 125 µl freezing solution and pipette up and down 10 times.

C. Transfer the 150 µl cell solution to a cryotube.

D. Add 350 µl freezing solution to the 150 µl cell solution.

E. Place the cryotubes in a cryogenic box and transfer to -80°C until further use.

Note At this step, cells can be kept in -80°C storage for 3 months.

Thawing of frozen cells

F. Put the cryotubes in a 37°C bath for up to 5 minutes (confirm that the cell solution has thawed).

G. Transfer the thawed 500 µl cell solution to a 1.5 ml Eppendorf tube.

   Add 500 µl **Wash buffer** to the empty cryotube and pipette up and down 10 times to wash any remaining cells.

H. Transfer the 500 µl wash to the Eppendorf tube. Total of 1000 µl in each tube.

I. Centrifuge at 700 rcf for 4 min at RT.

J. Remove 950 µl supernatant without disturbing the cell pellet, leaving behind 50 µl.

K. Add 950 µl **Wash buffer** and pipette up and down 10 times.

L. Centrifuge at 700 rcf for 4 min at RT.

M. Remove 950 µl supernatant without disturbing the cell pellet, leaving behind 50 µl.

N. Add 100 µl 1x PBS to resuspend the pellet.

O. Optional: Determine the cell concentration after thawing, using either hemocytometer or other cell counting device (e.g. Countess II Automated Cell Counter).

Note At this step, cells can be kept at +4°C storage for 24 hours.

STEP 2 - Antibody-Oligo Conjugate (AOC) binding

Step 2 contains binding of Antibody-oligo conjugates (AOCs) to protein targets, and their stabilization on cells using a secondary antibody. Cells are counted and diluted at the end of this part of the workflow prior to starting the Molecular Pixelation steps.

Note From this step and forward, make sure cells become resuspended during pipette mixing steps by mixing vigorously. Too gentle pipette-mixing may result in cell aggregate formation.

AOC binding

A. Centrifuge the vial containing **AOC panel** solution at 17000 rcf for 10 min at RT or 4°C, to pull down any aggregates to the bottom of the **AOC panel** vial.

B. While centrifuging the AOC panel, centrifuge the cell suspensions in another centrifuge at 700 rcf for 4 min at RT.

C. Carefully discard about 125 µl (less if cells were used for counting) sample supernatant without disturbing the cell pellet, leaving behind 25 µl supernatant to preserve the pellet.

D. Add 25 µl of the centrifuged **AOC panel** solution to each 25 µl reaction, to a final volume of 50 µl, and pipette up and down 10 times. Make sure to avoid aspirating near the bottom of the centrifuged **AOC panel** vial.

E. Incubate for 45 min at 4°C.

F. Add 100 µl **Wash Buffer** on top of the 50 µl cell suspension and pipette up and down 10 times.

G. Centrifuge at 700 rcf for 4 min at RT.

H. Carefully discard 125 µl supernatant without disturbing the cell pellet, leaving behind 25 µl supernatant to preserve the pellet.

I. Add 125 µl **Wash Buffer** on top of the 25 µl cell suspension and pipette up and down 10 times.

J. Centrifuge at 700 rcf for 4 min at RT.

K. Carefully discard 125 µl of supernatant without disturbing the cell pellet, leaving behind 25 µl supernatant to preserve the pellet.

L. Repeat steps I-K 2 times, for a total of 4 washes (including F-H). It is important to perform in total 4 washes to completely remove any remaining unbound AOCs present in the solution.

M. Quick spin the **2nd Antibody** and pipette it up and down 5 times to mix.

N. Add 25 µl of **2nd Antibody** to the 25 µl cell pellet and pipette up and down 10 times.

O. Incubate for 30 min at 37°C.

P. Add 100 µl **Wash Buffer** on top of the 50 µl cell suspension and pipette up and down 10 times.

Q. Centrifuge at 700 rcf for 4 min at RT.

R. Carefully discard 125 µl supernatant without disturbing the cell pellet, leaving behind 25 µl supernatant to preserve the pellet.

S. Add 125 µl **Wash Buffer** on top of the 25 µl cell suspension and pipette up and down 10 times.

T. Centrifuge at 700 rcf for 4 min at RT.

U. Carefully discard 125 µl supernatant without disturbing the cell pellet, leaving behind 25 µl supernatant to preserve the pellet.

V. Resuspend the cell pellet in 75 µl of 1xPBS and pipette up and down 10 times. Total of 100 µl in each tube.

W. Determine the cell concentration for each reaction using either hemocytometer or other cell counting device (e.g Countess II Automated

Cell Counter). Mix by pipetting up and down 10 times before taking an aliquot for counting.

Note Make sure to not use too big of a portion of the reaction for counting, as that may result in a deficit of cell input to MPX (20 000 cells).

Note At this step, cells can be stored at 4°C in 1xPBS for up to 2 days before proceeding with the next steps of the protocol.

STEP 3.1 - Molecular Pixelation (Pixelation A and B)

In this step of the protocol, a series of DNA hybridization and enzymatic treatments are performed to incorporate DNA-pixel barcodes onto AOC oligos bound to the cells, forming the amplicons that can be amplified by PCR and finally sequenced. Reminder: quick spin all reagents and pipette each reagent tube up and down 5 times to mix before preparing the Master mix.

DNA-Pixel A hybridization

Note Recommended starting point is to load 20 000 cells per sample in 1xPBS to a total volume of 25 µl.

Note The protocol can be paused for up to 1h between the steps Pixel A remover and DNA-Pixel B hybridization.

A. Dilute cells in fresh PCR tubes to 800 cells/µl in 1x PBS for a total of 25 µl (20 000 cells in total per reaction).

Note If the cell concentration is lower than 800 cells/µl, centrifuge reaction at 700 rcf for 4 min at RT and remove e.g. 50% of the volume to get 2x original concentration.

B. Prepare **Pixel A** hybridization master mix as indicated in Table 6. directly before use by combining the reagents in the order listed below, and maintain at RT.

Table 6. **Pixel A** hybridization master mix preparation.

Component	1x rxn + 10% extra	4x rxn + 10% extra	8x rxn + 10% extra	_ x rxn + 10% extra
Pixel A buffer	16.5 µl	66 µl	132 µl	
Pixel A	11 µl	44 µl	88 µl	
Total	27.5 µl	110 µl	220 µl	

C. Quick spin the **Pixel A** hybridization master mix and pipette up and down 5 times to mix.

D. Dispense 25 µl **Pixel A** hybridization master mix to each 25 µl reaction and pipette up and down 10 times. Total of 50 µl in each tube.

E. Incubate the reactions in a thermal cycler for 15 min at 55°C.

F. Remove the reactions from the thermal cycler, add 100 µl **Wash Buffer** to each reaction at RT and pipette up and down 10 times. Total of 150 µl in each tube.

G. Centrifuge at 700 rcf for 4 min at RT.

H. Carefully discard 125 µl of supernatant without disturbing the cell pellet, leaving behind 25 µl supernatant to preserve the pellet.

I. To each reaction, add 125 µl of **Wash Buffer** and pipette up and down 10 times.

J. Centrifuge at 700 rcf for 4 min at RT.

K. Carefully discard 125 µl of supernatant without disturbing the cell pellet, leaving behind 25 µl supernatant to preserve the pellet.

Gap-fill ligation step 1

A. Thaw the **Gap-fill Buffer** and prepare **Gap-fill** master mix as indicated in Table 7. directly before use by combining the reagents in the order listed below and maintain at RT. Keep the **Gap-fill Buffer** on ice or at 4°C, until used at the second Gap-fill ligation step 2 of the protocol (put the enzymes back at -20°C until second Gap-fill ligation step).

Table 7. **Gap-fill** master mix preparation.

Component	1x rxn + 10% extra	4x rxn + 10% extra	8x rxn + 10% extra	_ x rxn + 10% extra
Gap-fill Buffer	25.3 µl	101.2 µl	202.4 µl	
Gap-fill Ligase	1.1 µl	4.4 µl	8.8 µl	
Gap-fill Polymerase	1.1 µl	4.4 µl	8.8 µl	
Total	27.5 µl	110 µl	220 µl	

B. Quick spin the **Gap-fill** hybridization master mix and pipette up and down 5 times to mix.

C. Dispense 25 µl **Gap-fill** hybridization master mix to each 25 µl reaction and pipette up and down 10 times. Total of 50 µl in each tube.

D. Incubate the reactions in a thermal cycler for 20 min at 37°C.

E. Add 100 µl of **Wash Buffer** to each reaction at RT and pipette up and down 10 times. Total of 150 µl in each tube.

F. Centrifuge at 700 rcf for 4 min at RT.

G. Carefully discard 125 µl of supernatant without disturbing the cell pellet, leaving behind 25 µl supernatant to preserve the pellet.

Pixel A remover

A. Prepare **Pixel A Remover** master mix according to Table 8. directly before use by adding the reagents in the order listed below and maintain at RT.

Table 8. **Pixel A Remover** master mix preparation.

Component	1x rxn + 10% extra	4x rxn + 10% extra	8x rxn + 10% extra	_ x rxn + 10% extra
Wash Buffer	26.4 µl	105.6 µl	211.2 µl	
USER enzyme	1.1 µl	4.4 µl	8.8 µl	
Total	27.5 µl	110 µl	220 µl	

B. Quick spin the **Pixel A Remover** master mix and pipette up and down 5 times to mix.

C. Dispense 25 µl **Pixel A Remover** master mix to each 25 µl sample and pipette up and down 10 times. Total of 50 µl in each tube.

D. Incubate the samples in a thermal cycler for 15 min at 37°C.

E. Add 100 µl of **Wash Buffer** to each sample at RT and pipette up and down 10 times. Total of 150 µl in each tube.

F. Centrifuge at 700 rcf for 4 min at RT.

G. Carefully discard 125 µl of supernatant without disturbing the cell pellet, leaving behind 25 µl supernatant to preserve the pellet.

Note If pausing for up to 1 h at this step, resuspend the pellet by pipetting up and down 10 times before putting the samples at 4°C.

DNA-pixel B hybridization

A. Prepare **Pixel B** hybridization master mix as indicated in Table 9. shortly before use by adding the reagents in the order listed below and maintain at RT.

Table 9. **Pixel B** hybridization master mix preparation.

Component	1x rxn + 10% extra	4x rxn + 10% extra	8x rxn + 10% extra	_ x rxn + 10% extra
Pixel B buffer	16.5 µl	66 µl	132 µl	
Pixel B	11 µl	44 µl	88 µl	
Total	27.5 µl	110 µl	220 µl	

B. Quick spin the **Pixel B** hybridization master mix and pipette up and down 5 times to mix.

C. Dispense 25 µl **Pixel B** hybridization master mix to each 25 µl sample and pipette up and down 10 times. Total of 50 µl in each tube.

D. Incubate the samples in a thermal cycler for 15 min at 55°C.

E. Add 100 µl **Wash Buffer** to each sample at RT and pipette up and down 10 times. Total of 150 µl in each tube.

F. Centrifuge at 700 rcf for 4 min at RT.

G. Carefully discard 125 µl of supernatant without disturbing the cell pellet, leaving behind 25 µl of supernatant to preserve the pellet.

H. To each sample, add 125 µl **Wash Buffer** and pipette up and down 10 times.

I. Centrifuge at 700 rcf for 4 min at RT.

J. Carefully discard 125 µl of supernatant without disturbing the cell pellet, leaving behind 25 µl of supernatant to preserve the pellet.

Gap-fill ligation step 2

A. Prepare **Gap-fill** master mix as indicated in Table 10. directly before use by adding the reagents in the order listed below and maintain at RT. Return potential remainders of the Gap-fill Buffer and enzymes to -20°C storage.

Table 10. **Gap-fill** master mix preparation.

Component	1x rxn + 10% extra	4x rxn + 10% extra	8x rxn + 10% extra	_ x rxn + 10% extra
Gap-fill Buffer	25.3 µl	101.2 µl	202.4 µl	
Gap-fill Ligase	1.1 µl	4.4 µl	8.8 µl	
Gap-fill Polymerase	1.1 µl	4.4 µl	8.8 µl	
Total	27.5 µl	110 µl	220 µl	

B. Quick spin the **Gap-fill** master mix and pipette up and down 5 times to mix.

C. Dispense 25 µl **Gap-fill** master mix to each 25 µl sample and pipette up and down 10 times. Total of 50 µl in each tube.

D. Incubate the samples in a thermal cycler for 20 min at 37°C.

E. Add 100 µl of **Wash Buffer** to each sample at RT and pipette up and down 10 times. Total of 150 µl in each tube.

F. Centrifuge at 700 rcf for 4 min at RT.

G. Carefully discard 125 µl of supernatant without disturbing the cell pellet, leaving behind 25 µl supernatant to preserve the pellet.

H. To each sample, add 125 µl of **Wash Buffer** and pipette up and down 10 times.

I. Centrifuge at 700 rcf for 4 min at RT.

J. Carefully discard 125 µl of supernatant without disturbing the cell pellet, leaving behind 25 µl supernatant to preserve the pellet.

K. Add 50 µl **Wash Buffer** to each sample at RT and pipette up and down 10 times. Total of 75 µl in each tube.

Note At this step, cells can be stored at 4°C for up to 16 hours before proceeding with the next steps of the protocol.

STEP 3.2 - Molecular Pixelation (Exonuclease and PCR)

Exonuclease treatment

A. Count cells using a hemocytometer or single-use counting chamber. To ensure accuracy, count cells in replicates (2 reproducible counts). See *2. General Guidelines and Information: cell counting considerations* for more detailed information regarding cell counting.

B. For each sample, transfer 300 to 1000 cells (depending on experimental set-up) in **Wash Buffer** to a final volume of 7 µl, in new PCR tubes. See *Appendix 1* for an example table.

Note If the cell concentration is lower than 143 cells/µl, centrifuge sample at 700 rcf for 4 min at RT and remove e.g. 50% of the volume to get 2x original concentration.

C. Prepare **Exonuclease** master mix as indicated in Table 11. directly before use by adding the reagents in the order listed below, pipette up and down 10 times and maintain at RT until use.

Table 11. **Exonuclease** master mix preparation.

Component	1x rxn + 10% extra	4x rxn + 10% extra	8x rxn + 10% extra	_ x rxn + 10% extra
Gap-fill buffer	7.7 µl	30.8 µl	61.6 µl	
Exonuclease	1.1 µl	4.4 µl	8.8 µl	
Total	8.8 µl	35.2 µl	70.4 µl	

D. Dispense 8 µl **Exonuclease** master mix to each 7 µl cell sample containing cells, for a total of 15 µl in each tube.

E. Incubate in a thermal cycler at 37°C for 30 min, followed by 75°C for 10 min (Table 12).

Proceed to PCR.

Table 12. Incubation protocol for **Exonuclease** treatment.

Lid temperature	Reaction Volume	Run Time
105°C	15 µl	40 min
**Step**	**Temperature**	**Duration**
1	37°C	00:30:00
2	75°C	00:10:00
3	4°C	Hold

Sample index PCR

Sample indexes are introduced during PCR, allowing for pooling and sequencing multiple reactions in the same sequencing run. The reagent kit is supplied with 8 different sample indexes. Please see Paragraph 4 “General Guidelines & Information" in this document, for more detailed description and considerations related to sample indexing.

Note Make sure to use different index primers for each sample and note which sample index was used for each sample.

A. Pre-program a PCR system with the PCR program denoted in Table 13.

B. Thaw the **PCR Master Mix** vial completely prior to use. Quick spin the **PCR Master Mix** and pipette up and down 5 times to mix.

C. Add 5 µl of PCR index primer to each 15 µl exonuclease treated sample.

D. Add 20 µl of **PCR Master Mix** and pipette up and down 10 times to mix. Total of 40 µl per sample.

E. Perform PCR using the following PCR program (Table 13.).

Table 13. PCR program.

Lid temperature	Reaction Volume	Run Time	
105°C	40 µl	~ 42 min	
**Step**	**Temperature**	**Time**	**Number of cycles***
1	98°C	00:01:00	1
2	98°C	00:00:15	1000 cells: 13 cycles<br>500 cells: 14 cycles<br>300 cells: 15 cycles
3	60°C	00:00:30	
4	72°C	00:00:40	
5	72°C	00:05:00	1
6	4°C	Hold	

* Adjust number of PCR cycles with number of cells input to PCR reaction.

Note The PCR products can be stored at 4°C for up to 24 hours or at -20°C for long term storage.

Note **Quality control** (QC) can be performed at this step, using an aliquot of the PCR product for gel electrophoresis. QC can also be performed after the AMPure beads clean-up, using instruments such as BioAnalyzer™, Tapestation™ or Fragment Analyzer™. See section *STEP 4 - Quantification and quality control of purified PCR products* for more information.

STEP 4 - NGS preparation

AMPure XP beads clean-up

The PCR products are purified using Ampure XP SPRI beads prior to NGS sequencing to remove primers, salts, dNTPs etc. Two consecutive rounds of AMPure cleanup is performed to ensure all primers have been removed, as any remaining primers will negatively affect sequencing results.

Third party Consumables and hardware required:

- AMPure XP (Beckman Coulter, cat. no. A63880)
- Invitrogen™ DynaMag™-96 Side Magnet (ThermoFisher, cat. no. 12331D)
- TE Buffer (10 mM Tris-Acetate pH 8.0, 1 mM EDTA) for DNA elution
- Freshly prepared 70% ethanol

Note Place Agencourt AMPure XP beads at RT for 30 min before use, as recommended by Beckman Coulter

Note Always use freshly prepared 70% ethanol, as recommended by Beckman Coulter. Prepare 1 ml (720 µl + extra) 70% ethanol per sample.

A. Vortex the Agencourt AMPure XP bottle to resuspend any magnetic particles that may have settled.

B. For each sample, transfer 30 µl of PCR product to fresh PCR tubes. The remaining volume can be stored at -20°C.

C. Add 45 µl (1.5x bead:sample ratio) of resuspended Ampure bead mixture to each sample. Pipette mix 10 times to form a homogenous mixture.

D. Incubate for 5 min at RT.

E. Place the PCR tubes on a PCR tube magnet and incubate for 2 min, until the beads have settled against the tube wall.

F. Carefully remove the supernatant, without disturbing beads.

G. Add 180 µl of 70% ethanol to the beads pellet while on the magnet and incubate for 30 sec.

H. Carefully remove the supernatant, without disturbing beads.

I. Add 180 µl of 70% ethanol to the beads pellet while on the magnet and incubate for 30 sec.

J. Carefully remove the supernatant, without disturbing beads. Try to remove as much supernatant as possible.

K. Completely remove the residual ethanol and air-dry beads with lid open until any remaining ethanol has evaporated.

Note Avoid over-drying the beads as this can negatively affect DNA recovery. The dried patch of beads will show some cracks if over-dried.

L. Remove the PCR tubes from the magnet.

M. Resuspend dried beads in 30 µl of TE Buffer and incubate for 2 min at room temperature to elute the PCR product from the beads.

N. Place the PCR tube on the magnet and incubate for 2 min, until the beads have settled against the tube wall.

Note Do not discard the supernatant in step O. as it contains the eluted sample.

O. Carefully transfer the 30 µl supernatant to new PCR tubes, without disturbing beads.

AMPure cleanup round 2: Repeat steps A-L for a **total of two cleanup rounds**. It is important to perform two rounds of cleanup to remove all PCR primers. Proceed with steps P-R to perform the second elution in 20 µl of TE Buffer.

P. Resuspend dried beads in 20 µl of TE Buffer and incubate for 2 min at room temperature to elute the PCR product from the beads.

Q. Place the PCR tube on the magnet and incubate for 2 min, until the beads have settled against the tube wall.

Note Do not discard the supernatant in step R. as it contains the eluted sample.

R. Carefully transfer the 20 µl supernatant to new PCR tubes, without disturbing beads.

The purified PCR products can either be prepared for sequencing directly, or stored at -20°C until use.

Appendix 1, MPX Buffer mix preparation

Gap-fill buffer:

Component	Concentration	
rCutSmart	2.17	x
NAD+	1.09	mM
dNTPs	0.22	mM

10x Hyb. Buffer

component	Concentration	
NaCl	3000	mM
MgCl2	150	mM
Tris buffer (pH8)	200	mM
Tween20	0.5	%

Pixel A buffer

Component	Concentration	
GPA oligo	6.67	uM
Hyb. buffer	3.33	x
ssDNA	3.33	mg/ml

Pixel B buffer

Component	Concentration	
GPB oligo	6.67	uM
Hyb. buffer	3.33	x
ssDNA	3.33	mg/ml
