# Supplementary material for: Molecular pixelation: spatial proteomics of single cells by sequencing
Source: Nat Methods. 2024 May 8;21(6):1044–52. doi: 10.1038/s41592-024-02268-9 (PMC11166577; doi:10.1038/s41592-024-02268-9)
Supplement: Supplementary file 2 — Reporting Summary [file 41592_2024_2268_MOESM2_ESM.pdf]

nature portfolio

Corresponding author(s): Simon Fredriksson

Last updated by author(s): February 13 2024

Reporting Summary

Nature Portfolio wishes to improve the reproducibility of the work that we publish. This form provides structure for consistency and transparency in reporting. For further information on Nature Portfolio policies, see our Editorial Policies and the Editorial Policy Checklist.

Statistics

For all statistical analyses, confirm that the following items are present in the figure legend, table legend, main text, or Methods section.

n/a	Confirmed	
☐	☒	The exact sample size (*n*) for each experimental group/condition, given as a discrete number and unit of measurement
☐	☒	A statement on whether measurements were taken from distinct samples or whether the same sample was measured repeatedly
☐	☒	The statistical test(s) used AND whether they are one- or two-sided<br>*Only common tests should be described solely by name; describe more complex techniques in the Methods section.*
☒	☐	A description of all covariates tested
☐	☒	A description of any assumptions or corrections, such as tests of normality and adjustment for multiple comparisons
☐	☒	A full description of the statistical parameters including central tendency (e.g. means) or other basic estimates (e.g. regression coefficient) AND variation (e.g. standard deviation) or associated estimates of uncertainty (e.g. confidence intervals)
☐	☒	For null hypothesis testing, the test statistic (e.g. *F*, *t*, *r*) with confidence intervals, effect sizes, degrees of freedom and *P* value noted<br>*Give P values as exact values whenever suitable.*
☒	☐	For Bayesian analysis, information on the choice of priors and Markov chain Monte Carlo settings
☒	☐	For hierarchical and complex designs, identification of the appropriate level for tests and full reporting of outcomes
☐	☒	Estimates of effect sizes (e.g. Cohen's *d*, Pearson's *r*), indicating how they were calculated

Our web collection on statistics for biologists contains articles on many of the points above.

Software and code

Policy information about availability of computer code

Data collection	All data was generated in-house.
Data analysis	FASTQ files were processed with an in-house software Pixelator (v0.12.0; https://github.com/PixelgenTechnologies/pixelator). Downstream analysis was performed in R (v4.2.2) using broom (v1.0.2), rstatix (v0.7.1), Seurat (v4.3.0), SeuratObject (v4.1.3), tidygraph (v1.2.2), and tidyverse (v1.3.2) for analysis, and ggforce (v0.4.1), ggplot2 (v3.4.0), ggplotify (v0.1.0), ggpubr (v0.5.0), ggraph (v2.1.0), graphlayouts (v0.8.4), igraph (v1.3.5), patchwork (v1.1.2), pheatmap (v1.0.12), plotly (v4.10.1), viridis (v0.6.2), and viridisLite (v0.4.1) for data visualization.<br><br>All analysis code can be found at https://github.com/PixelgenTechnologies/pixelgen-MPX-paper

For manuscripts utilizing custom algorithms or software that are central to the research but not yet described in published literature, software must be made available to editors and reviewers. We strongly encourage code deposition in a community repository (e.g. GitHub). See the Nature Portfolio guidelines for submitting code & software for further information.

Data

Policy information about availability of data

All manuscripts must include a data availability statement. This statement should provide the following information, where applicable:

- Accession codes, unique identifiers, or web links for publicly available datasets
- A description of any restrictions on data availability
- For clinical datasets or third party data, please ensure that the statement adheres to our policy

The MPX raw read data and Pixelator 0.12 processed output can be downloaded from Pixelgen Technologies (https://software.pixelgen.com/datasets/). Datasets are granted under a Creative Commons Attribution (https://creativecommons.org/licenses/by/4.0/legalcode) license.

Human research participants

Policy information about studies involving human research participants and Sex and Gender in Research.

Reporting on sex and gender	Data on neither sex nor gender were collected.
Population characteristics	no population studies were done
Recruitment	All PBMC samples were purchased from a Karolinska Hospital blood bank drawn from healthy volunteers with informed consent and withheld sample identity or other medical information.
Ethics oversight	*Identify the organization(s) that approved the study protocol.*

Note that full information on the approval of the study protocol must also be provided in the manuscript.

Field-specific reporting

Please select the one below that is the best fit for your research. If you are not sure, read the appropriate sections before making your selection.

☒ Life sciences ☐ Behavioural & social sciences ☐ Ecological, evolutionary & environmental sciences

For a reference copy of the document with all sections, see nature.com/documents/nr-reporting-summary-flat.pdf

Life sciences study design

All studies must disclose on these points even when the disclosure is negative.

Sample size	Since the study was primarily focused on method development, no calculations were carried out to determine the size of the biological samples. Previous experiments indicated that 500-1000 cells were sufficient to gain reproducible statistical significance in both abundance and spatial metrics for primary PBMC cell populations. For this methods development context, this range of cell input was therefore chosen.
Data exclusions	Cells were filtered to remove cells with few detected antibodies, and suspected antibody aggregates as outlined in Methods.
Replication	PBMC samples were processed in duplicates and cells were aggregated from both replicates for analysis. All replications were successful.
Randomization	Samples were randomly downsampled in silico for statistical tests.
Blinding	Blinding was not performed as this work is methods development.

Reporting for specific materials, systems and methods

We require information from authors about some types of materials, experimental systems and methods used in many studies. Here, indicate whether each material, system or method listed is relevant to your study. If you are not sure if a list item applies to your research, read the appropriate section before selecting a response.

Materials & experimental systems

n/a	Involved in the study	
☐	☒	Antibodies
☐	☒	Eukaryotic cell lines
☒	☐	Palaeontology and archaeology
☒	☐	Animals and other organisms
☒	☐	Clinical data
☒	☐	Dual use research of concern

Methods

n/a	Involved in the study	
☒	☐	ChIP-seq
☒	☐	Flow cytometry
☒	☐	MRI-based neuroimaging

Antibodies

Antibodies used

All antibodies purchased from Antibodies-online and used at 5ug/ml)

B2M B2M-02
BAFFR 11C1
CD102 CBR-IC2-2
CD11a HI111
CD11b ICRF44
CD11c Bu15
CD127 A019D5
CD137 4B4-1
CD14 61D3
CD150 A12 (7D4)
CD152 BNI3
CD154 24-31
CD158 NKVFS1
CD16 3G8
CD161 HP-3G10
CD162 KPL-1
CD163 GHI-61
CD18 TS1-18
CD19 SJ25-C1
CD197 G043H7
CD1d 51-1
CD2 TS1-8
CD20 2H7
CD200 OX-104
CD22 S-HCL-1
CD229 HLy9-25
CD244 C1.7
CD25 BC96
CD26 BA5b
CD27 LT27
CD274 2A3
CD278 C398-4A
CD279 EH12-2H7
CD29 3B6
CD314 1D11
CD32 FUN2
CD33 WM53
CD337 P30-15
CD35 E11
CD36 5-271
CD37 IPO-24
CD38 HIT2
CD3E UCHT1
CD4 OKT4
CD40 C40-1605
CD41 A2A9-6
CD43 MEM-59
CD44 F10-44-2
CD45 HI30
CD45RA HI100
CD45RB MEM55
CD47 B6H12-2
CD48 MEM-102
CD49D 9F10
CD5 UCHT2
CD50 MEM-171
CD52 HI186

CD53 MEM-53
CD54 1H4
CD55 F4-29D9
CD59 MEM-43
CD62P AK4
CD64 10.1
CD69 FN50
CD7 4H9
CD71 CY1G4
CD72 3F3
CD8 SK1
CD82 C33
CD84 CD84-1-21
CD86 IT2-2
CD9 MEM-61
HLA-ABC W6-32
HLA-DR L243
SIGLEC S7-7
TCRb MEM-262
ACTB 137CT26-1-1
mIgG1 MOPC-21
mIgG2a MOPC-173
mIgG2b MPC-11

Validation	All antibodies are mouse monoclonal and validated by the supplier antibodies-online.com for use in flow cytometry by their specific binding to respective target proteins found on a certain cell type in PBMC. Each antibody was revalidated by the authors in the same way on PFA fixed cells.

Eukaryotic cell lines

Policy information about cell lines and Sex and Gender in Research

Cell line source(s)	Purchased (Raji, Jurkat, Daudi) from DSMZ (www.dsmz.de)
Authentication	All cell lines used (Raji, Jurkat, Daudi) have been verified by flow cytometry.
Mycoplasma contamination	All cell lines (Raji, Jurkat, Daudi) have tested negative for mycoplasma contamination prior to the experiments.
Commonly misidentified lines (See ICLAC register)	None of the cell lines used (Raji, Jurkat, Daudi) are commonly misidentified according to the ICLAC register.
