# Supplementary figures and images for: Molecular pixelation: spatial proteomics of single cells by sequencing
Source: Nat Methods. 2024 May 8;21(6):1044–52. doi: 10.1038/s41592-024-02268-9 (PMC11166577; doi:10.1038/s41592-024-02268-9)

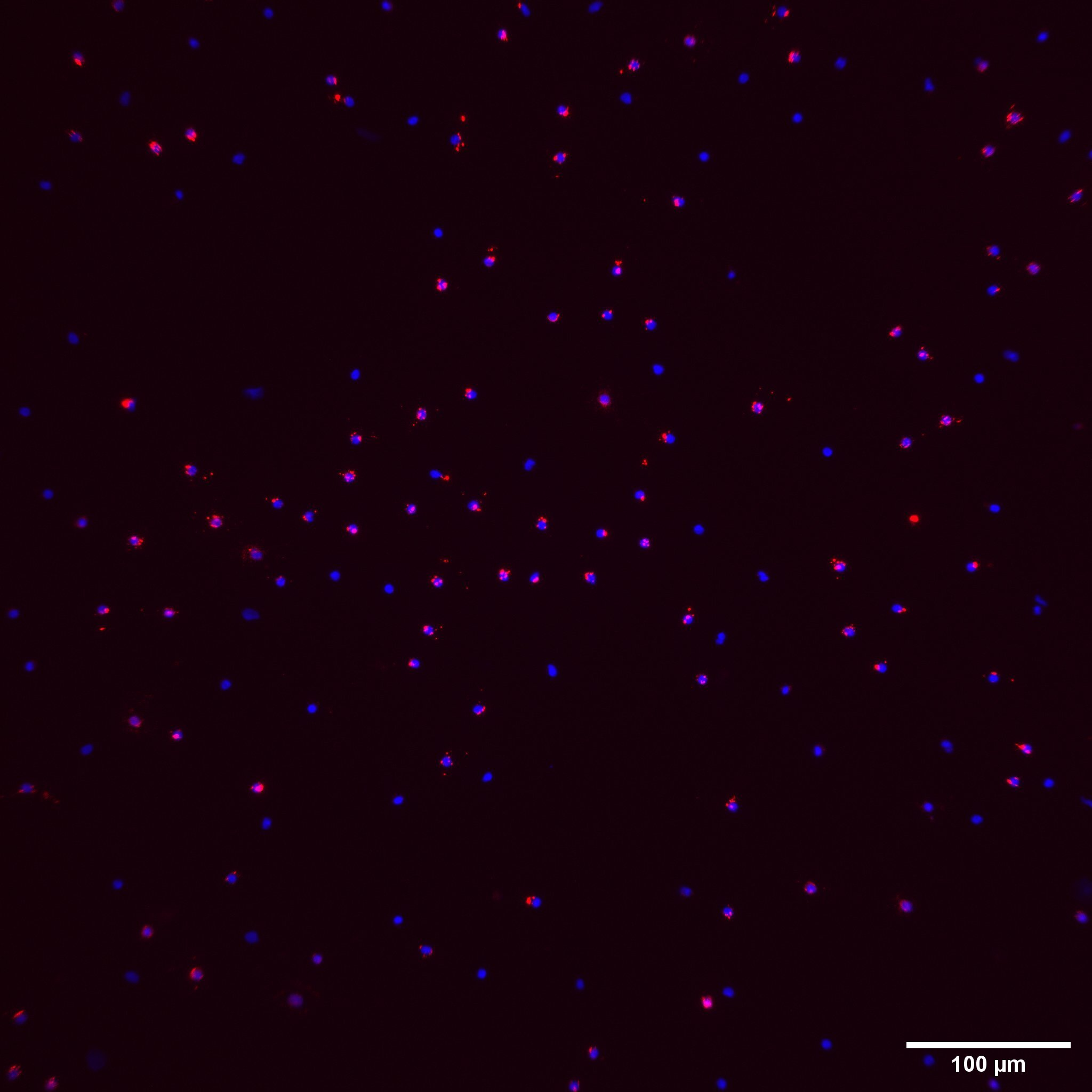

Supplement: Supplementary file 8 — Statistical source data and unprocessed immunofluorescence imaging of Fig. 3. [file 41592_2024_2268_MOESM8_ESM.zip › Source Figure 3/Fig3D_CD3_Capped_20x.tif]

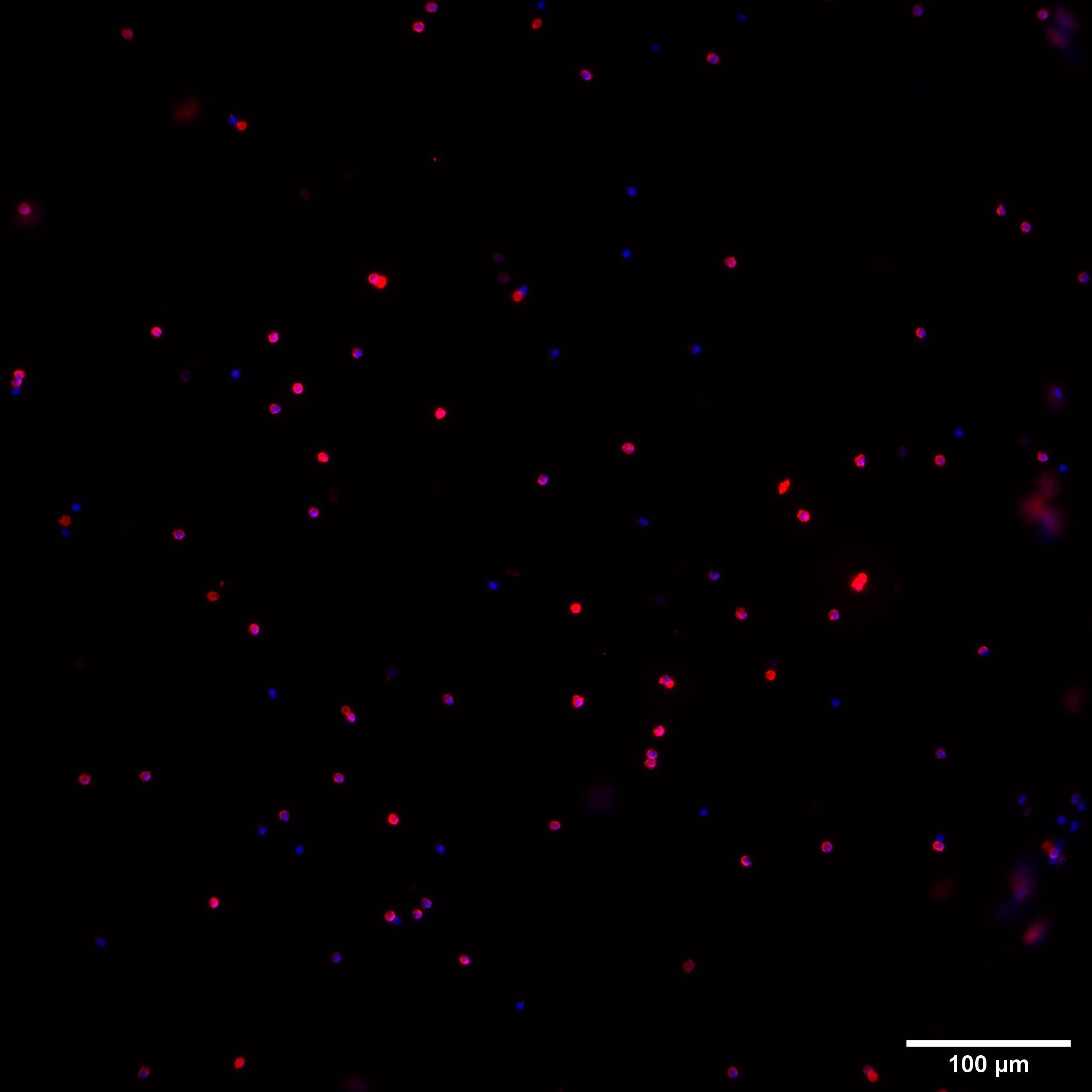

Supplement: Supplementary file 8 — Statistical source data and unprocessed immunofluorescence imaging of Fig. 3. [file 41592_2024_2268_MOESM8_ESM.zip › Source Figure 3/Fig3D_CD3_Control_20x.tif]

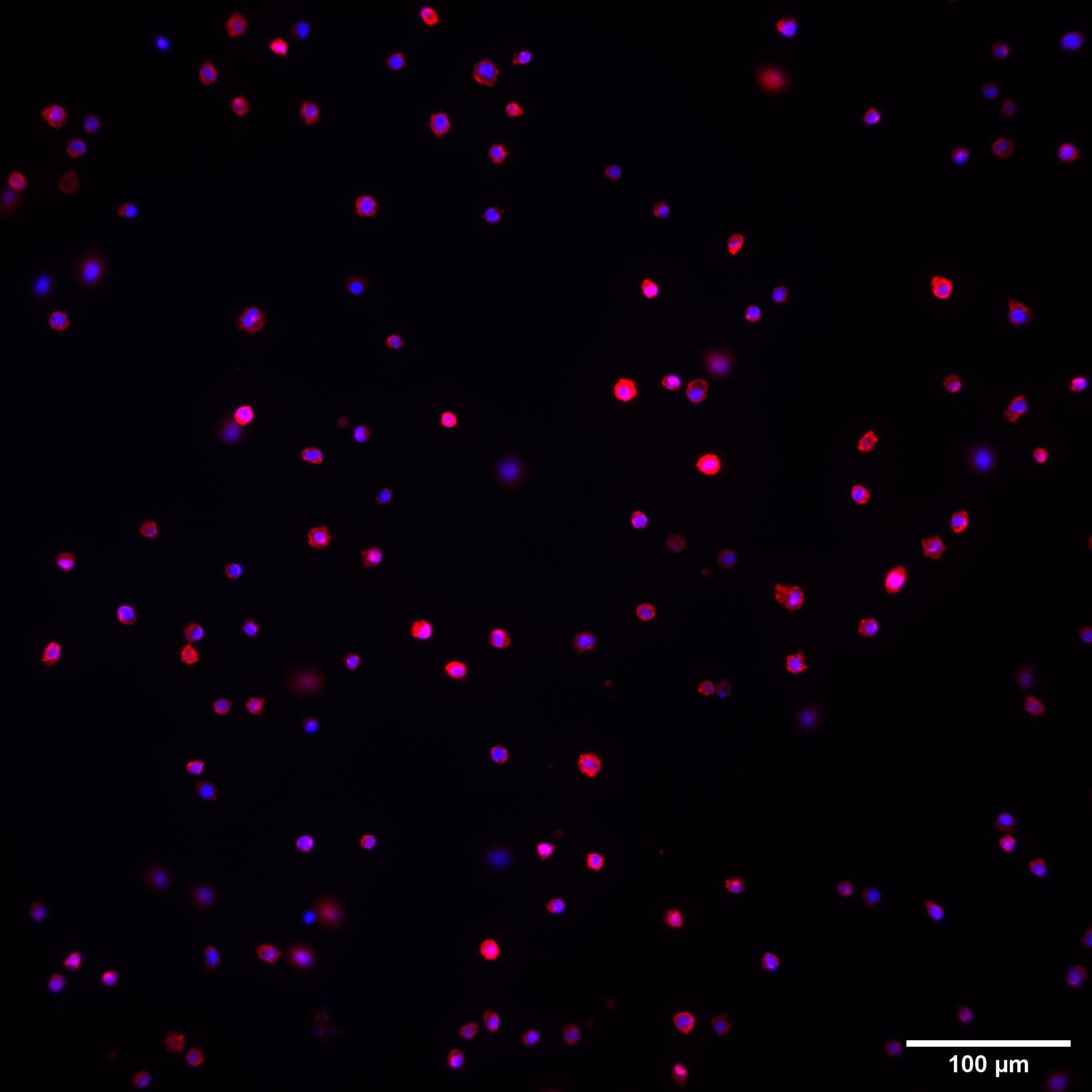

Supplement: Supplementary file 8 — Statistical source data and unprocessed immunofluorescence imaging of Fig. 3. [file 41592_2024_2268_MOESM8_ESM.zip › Source Figure 3/Fig3H_Raji_control_20x.tif]

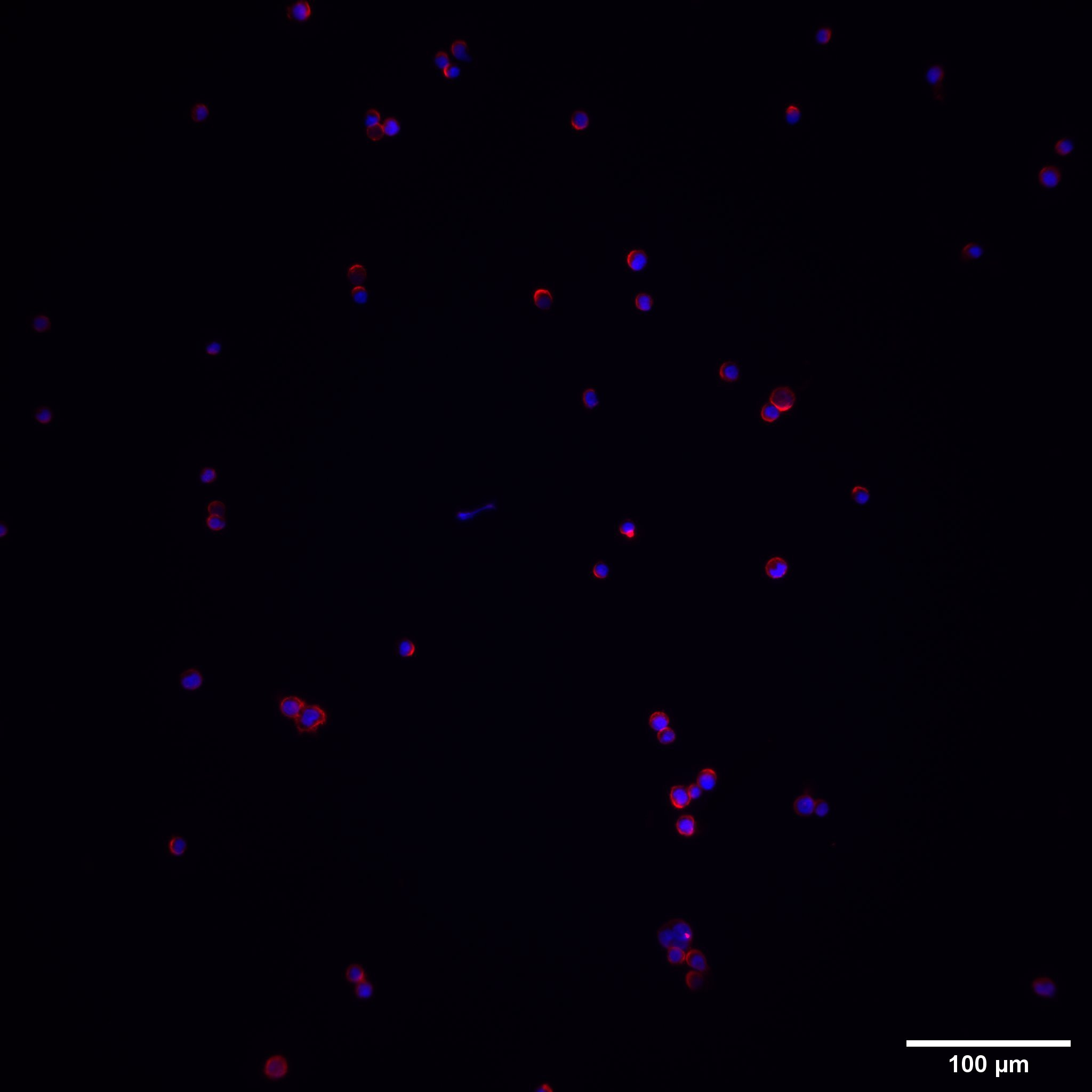

Supplement: Supplementary file 8 — Statistical source data and unprocessed immunofluorescence imaging of Fig. 3. [file 41592_2024_2268_MOESM8_ESM.zip › Source Figure 3/Fig3H_Raji_Ritux_20x.tif]

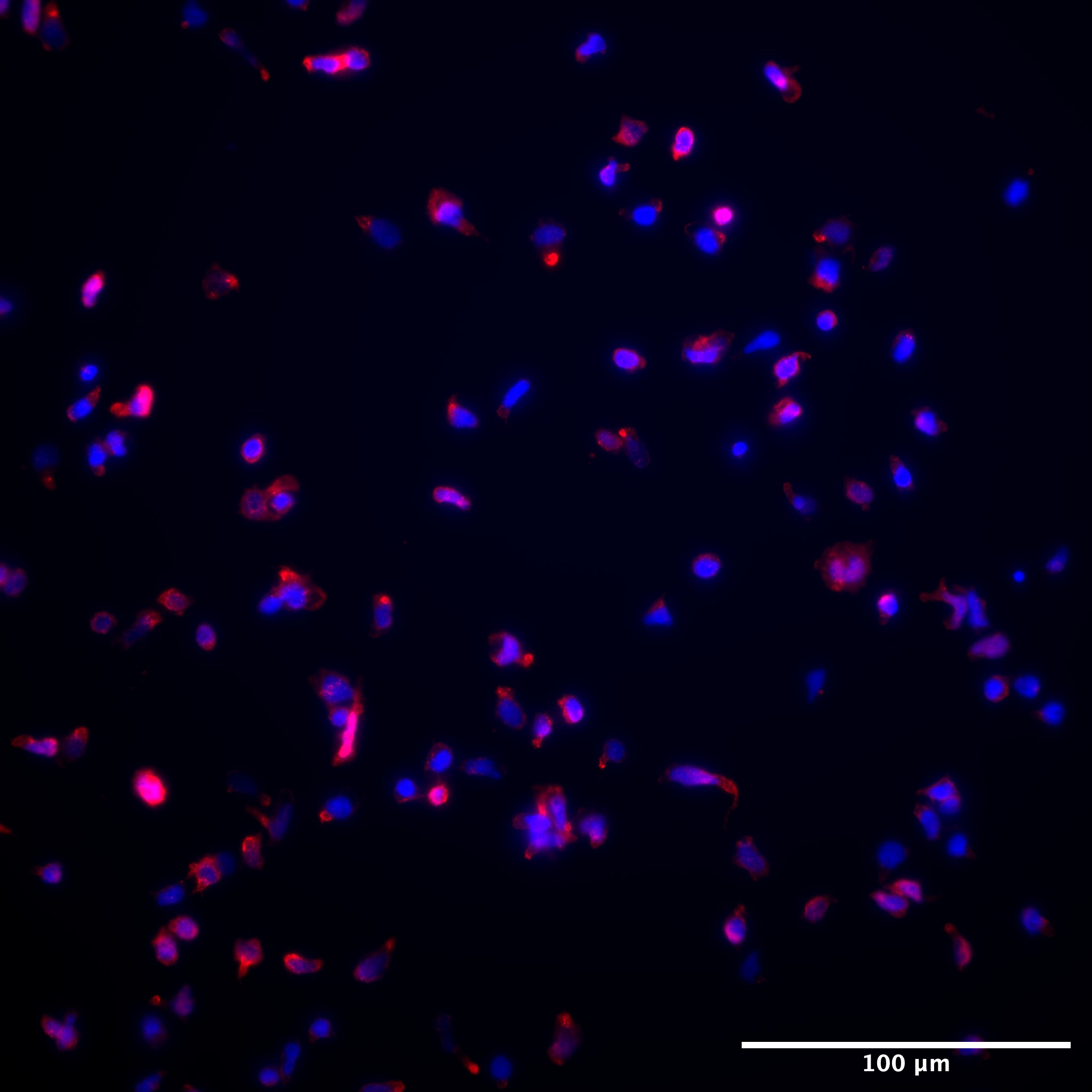

Supplement: Supplementary file 19 — Unprocessed immunofluorescence imaging of Extended Data fig. 9. [file 41592_2024_2268_MOESM19_ESM.zip › Source Extended Data Figure 9/Extended_Fig_9_CD162.tif]

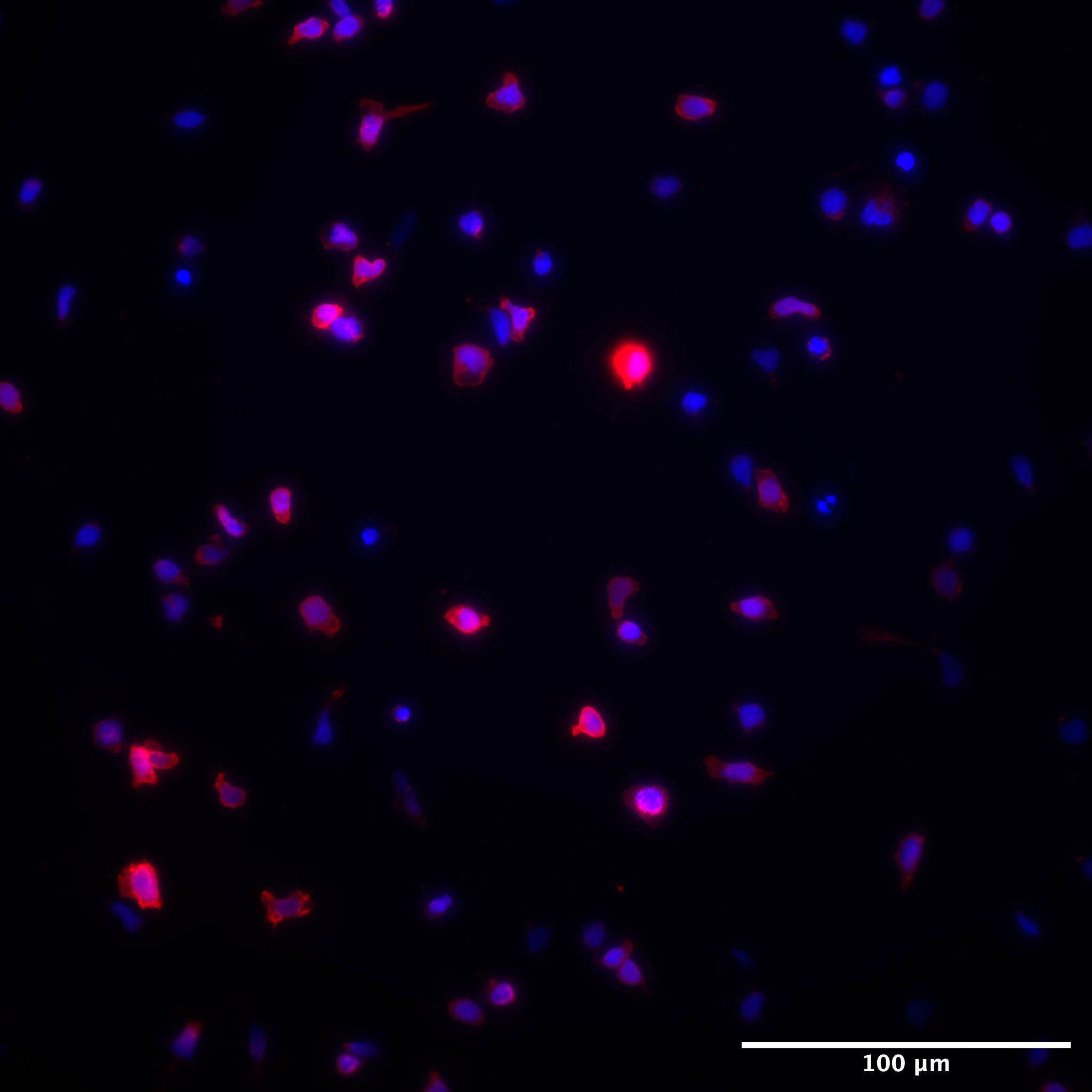

Supplement: Supplementary file 19 — Unprocessed immunofluorescence imaging of Extended Data fig. 9. [file 41592_2024_2268_MOESM19_ESM.zip › Source Extended Data Figure 9/Extended_Fig_9_CD3.tif]

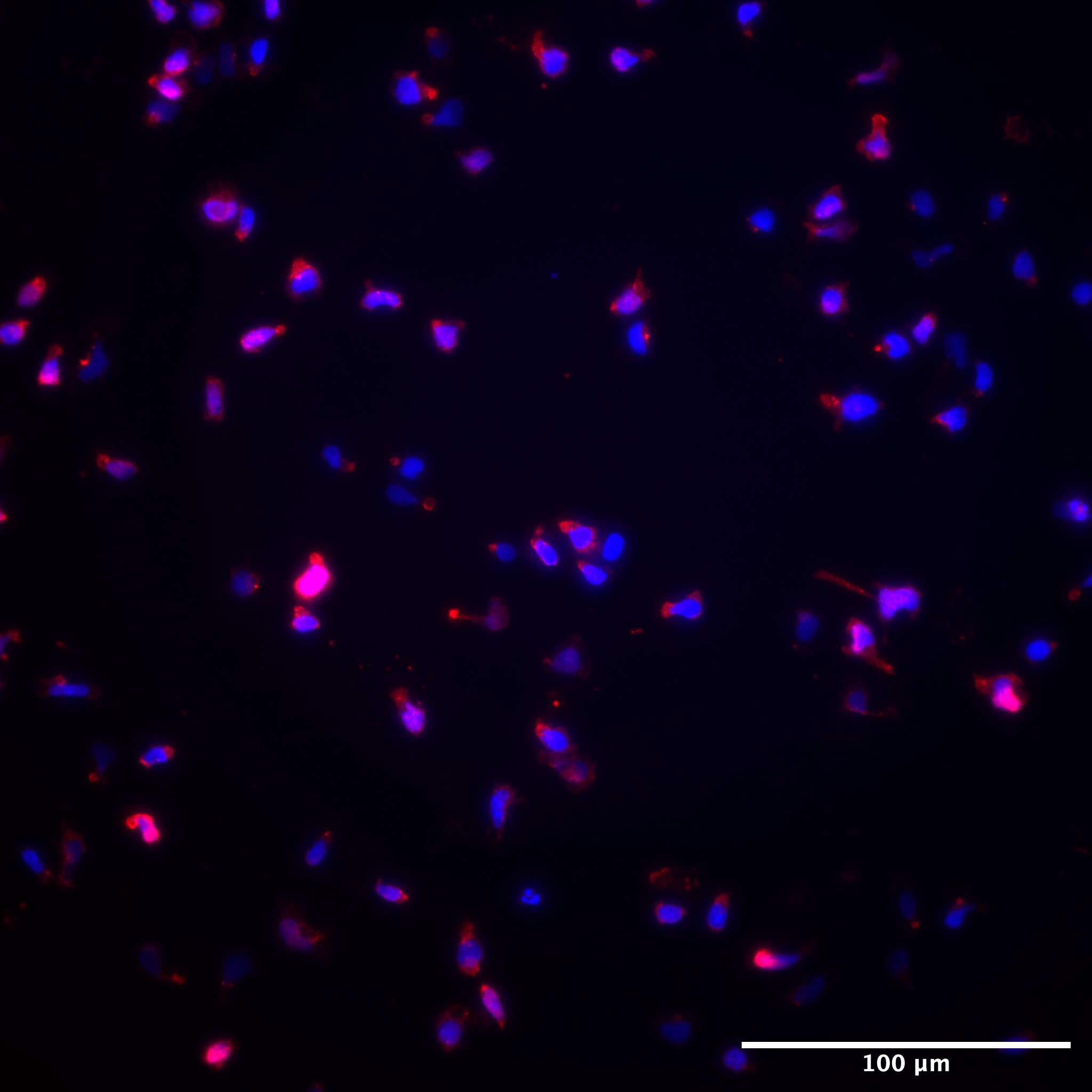

Supplement: Supplementary file 19 — Unprocessed immunofluorescence imaging of Extended Data fig. 9. [file 41592_2024_2268_MOESM19_ESM.zip › Source Extended Data Figure 9/Extended_Fig_9_CD37.tif]

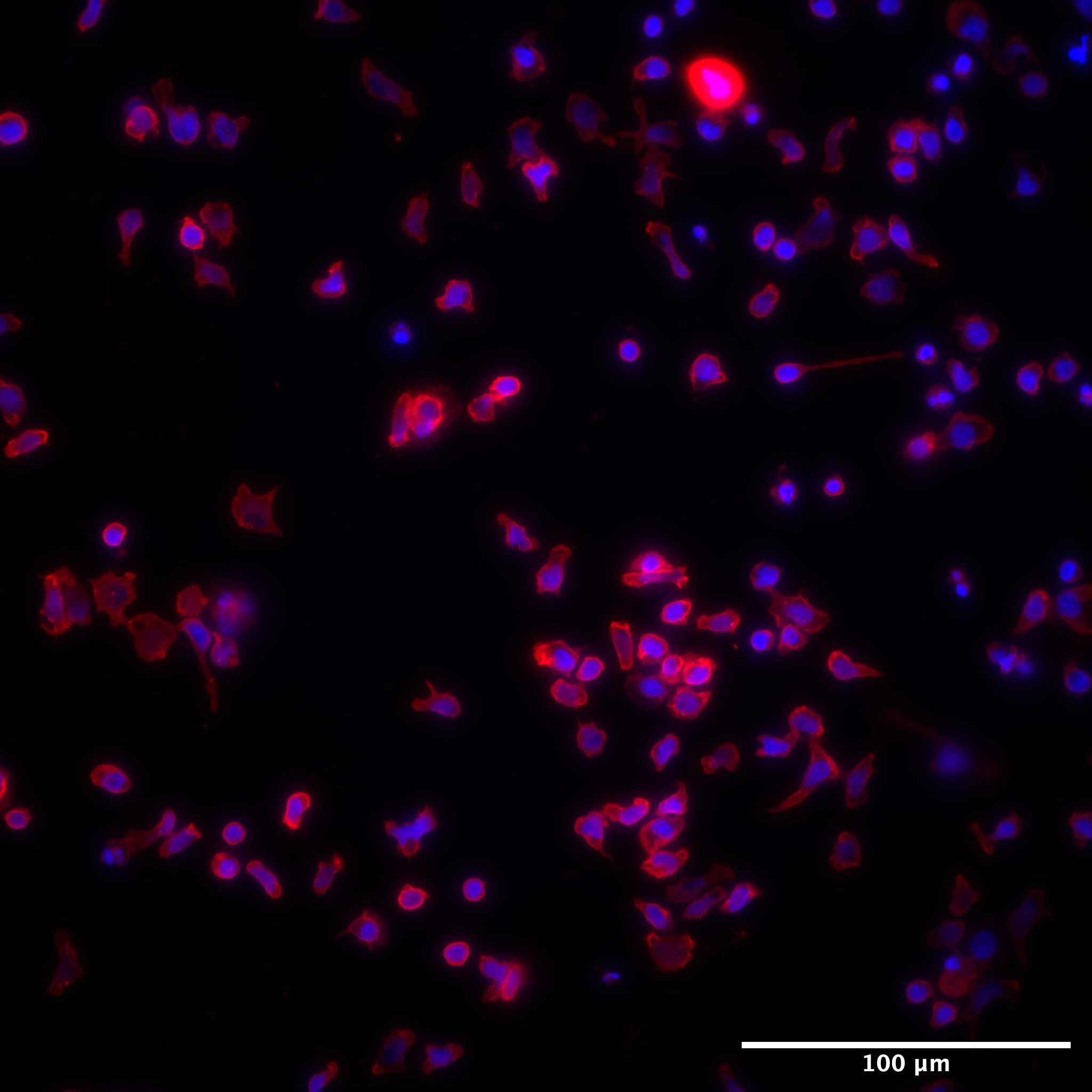

Supplement: Supplementary file 19 — Unprocessed immunofluorescence imaging of Extended Data fig. 9. [file 41592_2024_2268_MOESM19_ESM.zip › Source Extended Data Figure 9/Extended_Fig_9_CD45.tif]

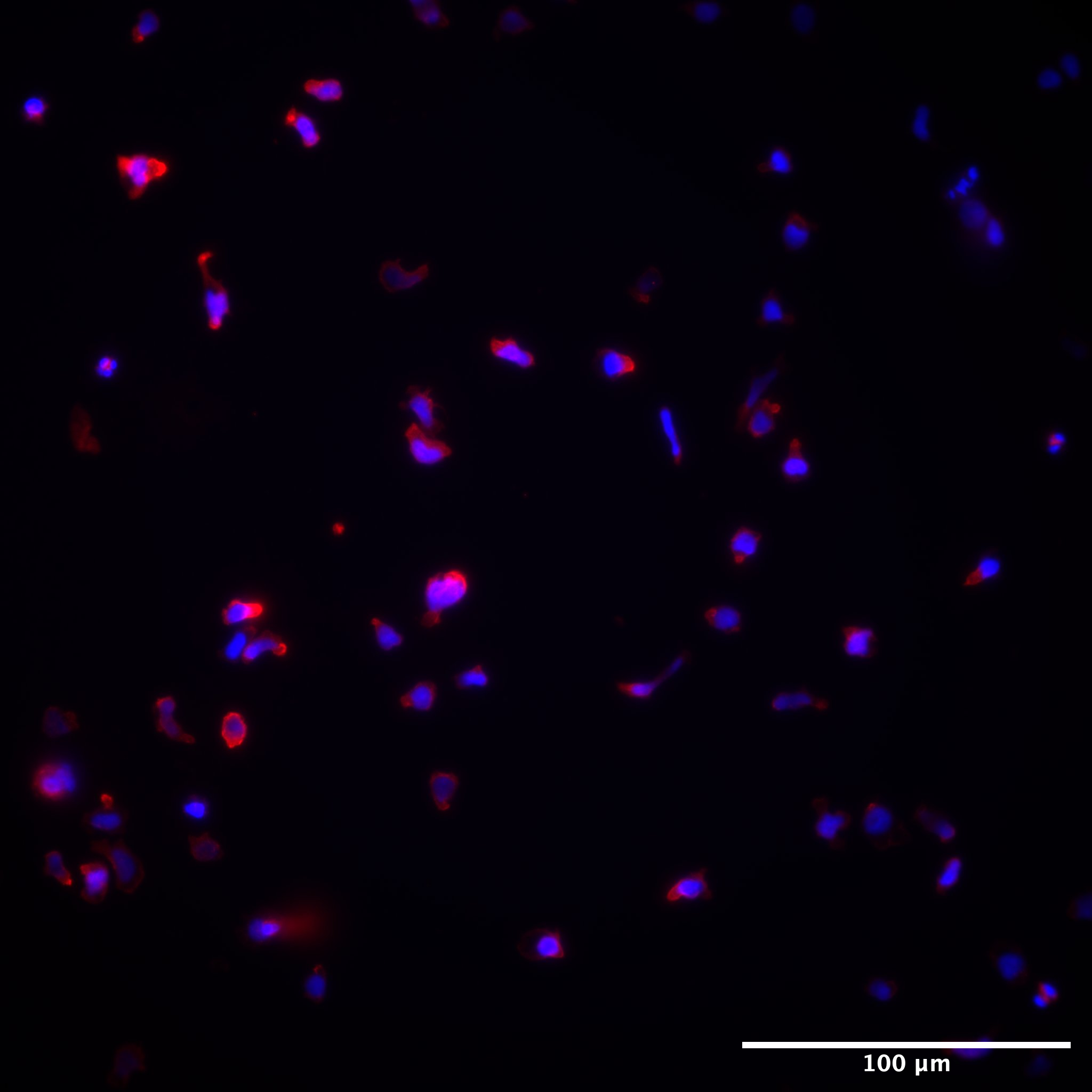

Supplement: Supplementary file 19 — Unprocessed immunofluorescence imaging of Extended Data fig. 9. [file 41592_2024_2268_MOESM19_ESM.zip › Source Extended Data Figure 9/Extended_Fig_9_CD50.tif]
